# Influence of *FKBP5* polymorphism and DNA methylation on structural changes of the brain in major depressive disorder

**DOI:** 10.1038/srep42621

**Published:** 2017-02-15

**Authors:** Kyu-Man Han, Eunsoo Won, Youngbo Sim, June Kang, Changsu Han, Yong-Ku Kim, Seung-Hyun Kim, Sook-Haeng Joe, Min-Soo Lee, Woo-Suk Tae, Byung-Joo Ham

**Affiliations:** 1 Department of Psychiatry, Korea University Anam Hospital, Korea University College of Medicine, Seoul, Republic of Korea; 2 Brain Convergence Research Center, Korea University Anam Hospital, Seoul, Republic of Korea; 3 Department of Biomedical Sciences, Korea University College of Medicine, Seoul, Republic of Korea; 4 Department of Psychiatry, Korea University Ansan Hospital, Korea University College of Medicine, Seoul, Republic of Korea; 5 Department of Psychiatry, Korea University Guro Hospital, Korea University College of Medicine, Seoul, Republic of Korea

## Abstract

A single nucleotide polymorphism of rs1360780 in the *FKBP5* gene is associated with a predisposition to developing major depressive disorder (MDD). We investigated the interactive effects of *FKBP5* rs1360780 allelic variants, DNA methylation, and the diagnosis of MDD on structural changes of the entire brain. One hundred and fourteen patients with MDD and eighty-eight healthy controls underwent T1-weighted structural magnetic resonance imaging and *FKBP5* rs1360780 genotyping, including DNA methylation of intron 7. We analyzed the volume of cortical and subcortical regions and cortical thickness using FreeSurfer. Significant genotype-by-diagnosis interactions were observed for volumes of the left pars triangularis, supramarginal gyrus, superior parietal lobule, right frontomarginal, and posterior midcingulate gyrus. The T allele was associated with significant volume reductions in these brain regions only in the MDD group except for the right posterior midcingulate gyrus. *FKBP5* DNA methylation showed a positive correlation with the thickness of the right transverse frontopolar gyrus in the C allele homozygote group. Our findings suggest that the *FKBP5* gene and its epigenetic changes could have influence on morphologic changes of several brain regions involved in emotion regulation, and that this process may be associated with the development of MDD.

The etiology of major depressive disorder (MDD) is characterized by a complex interplay between multiple genes and environmental factors^1^. A recent genome-wide association study reported two genome-wide significant loci contributing to predisposition to MDD^2^. Genetic variations involving monoaminergic neurotransmission^3^, neuroplasticity^4^,^5^, or the hypothalamic–pituitary–adrenal (HPA) axis^6^ influence structural and functional alterations of the neural network in patients with MDD. Accumulating evidence suggests that dysregulation of the HPA axis and stress/cortisol responsivity influences the predisposition to MDD^6^,^7^ and structural changes in brain regions involved in HPA axis regulation, such as the hippocampus and amygdala^8^,^9^,^10^. Several genetic variants are thought to be involved in the disturbed stress-regulatory hormonal system in MDD^8^,^11^.

The *FKBP5* gene encodes FK506 binding protein 51 (FKBP5), which is highly expressed after stress exposure, and the inhibitory role of FKBP5 in glucocorticoid receptor activity provides an ultra-short negative feedback loop for stress-induced increases in plasma cortisol^12^. It has been suggested that the rs1360780 risk allele of the *FKBP5* gene is associated with greater induction of FKBP5 by cortisol, compared to the non-risk allele^13^. The sequence containing the risk (T) allele leads to overexpression of FKBP5 following glucocorticoid receptor activation and dysregulated negative feedback on the stress-hormone system, with prolonged cortisol release after stress exposure^14^. Recent studies on *FKBP5* rs1360780 have suggested that the T allele is associated with a predisposition to MDD^15^. It has also been suggested that exposure to childhood trauma interacts with the T allele and leads to epigenetic changes, such as a reduced methylation of a second glucocorticoid response element located in intron 7 of the gene^16^. This results in an even stronger transcriptional activation of FKBP5 and disturbances to the HPA axis in addition to influencing the development of stress-related psychiatric disorders^16^.

Previous imaging studies on *FKBP5* rs1360780 have mainly investigated functional and structural alterations in the hippocampus or amygdala^8^,^11^,^17^,^18^. Recent evidence has suggested that dysregulation of the HPA axis in patients with MDD could cause structural changes to the cortico-limbic network involved in emotion regulation in regions other than the hippocampus or amygdala, such as the orbitofrontal cortex (OFC)^19^ or anterior cingulate cortex (ACC)^20^. Elevated transcript and protein levels of FKBP5 in the frontal cortex have been correlated with MDD in HIV-infected patients^21^, and *FKBP5* mRNA expression has been detected in various brain regions including the cerebral cortex in the adult mouse^22^. This evidence has led to investigations on the influence of the *FKBP5* genetic variant on structural changes of the entire brain, including the cortico-limbic network in MDD. However, few studies conducted on patients with MDD have explored the association between the rs1360780 allelic variant and structural changes of brain regions other than the hippocampus or amygdala. One study investigated structural changes to the whole brain of patients with MDD influenced by *FKBP5* genotype; however, this study only investigated microstructural changes of white matter tracts^23^. Also, although another study explored the effects of rs1360780 on cortical volume and integrity of white matter tracts throughout the brain, the study sample was drawn from a non-clinical population^24^.

A study by Höhne *et al*. has reported that a lifetime history of MDD and rs1360780 have significant interaction effects on epigenetic changes of intron 7 in the *FKBP5* gene^25^. Epigenetic changes to the *FKBP5* gene have come into the spotlight due to their involvement in the pathophysiology of psychiatric disorders^12^,^26^. However, there are as yet no brain imaging studies on *FKBP5* DNA methylation and MDD. Additionally, a recent meta-analysis with a large sample indicates that patients with MDD have thinner cortical gray matter in the OFC, the anterior and posterior cingulate, the insula, and the temporal lobe, and suggests that cortical thickness changes in MDD are robustly detectable findings^27^. Thus, there is an increased need for a comprehensive approach to elucidate the influence of *FKBP5* rs1360780 and DNA methylation on changes in cortical volumes and thickness of the entire brain in patients with MDD.

In this study, we aimed to investigate the interactive effects among *FKBP5* rs1360780 allelic variants, DNA methylation, and diagnosis of MDD on volume changes in cortical and subcortical regions of the entire brain and on cortical thickness. Our *a priori* hypotheses were as follows: 1) Significant interactive effects between *FKBP5* rs1360780 genotype and MDD diagnosis on gray matter volume and/or cortical thickness reductions in the cortico-limbic network will be observed. 2) Significant correlations between DNA methylation and gray matter volume and/or cortical thickness in the cortico-limbic network according to *FKBP5* rs1360780 risk allele and/or diagnosis of MDD will be observed.

## Results

### Demographic and genotype characteristics

Age, gender, education level, the 17-item Hamilton Depression Rating Scale (HDRS) score, duration of illness, allelic variant of *FKBP5* rs1360780, and the proportion of drug-naïve patients according to genotype are shown in Table 1. There were no significant differences between the two groups except for the HDRS score (t_(200)_ = 15.973, P < 0.001).

### *FKBP5* rs1360780 genotype and gray-matter volumes and cortical thickness

**Figure 1:**
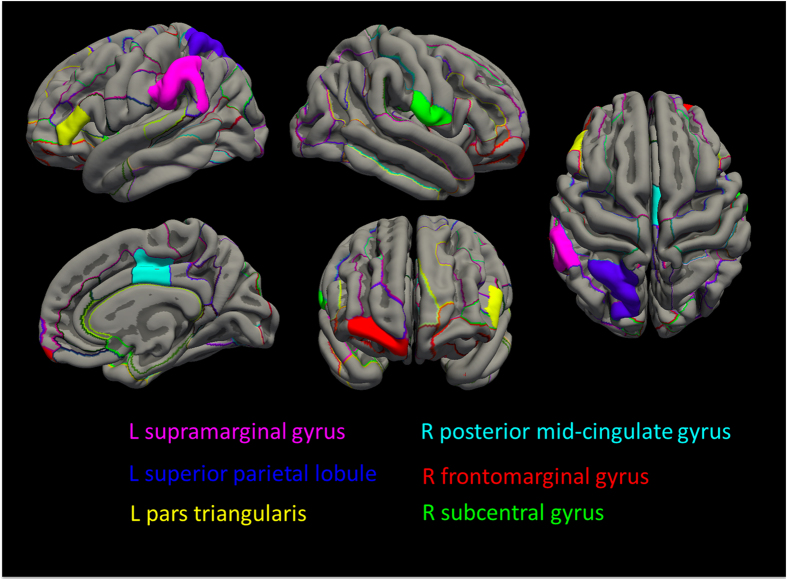
Schematic maps on the cortical regions with significant genotype-by-diagnosis interaction or genotype effect. The cortical parcellation criteria of the Destrieux atlas was applied. The significant interactive effect of the *FKBP5* rs1360780 genotype and major depressive disorder (MDD) diagnosis was observed in left pars triangularis (P_corr_ = 0.027), supramarginal gyrus (P_corr_ = 0.027), superior parietal lobule (P_corr_ = 0.017), and right frontomarginal (P_corr_ = 0.043) and posterior midcingulate gyrus (P_corr_ = 0.043). In all participants of both the MDD and healthy control groups, T allele was associated with a smaller volume in the right subcentral gyrus among the total participants (P_corr_ = 0.001) (R, right hemisphere; L, left hemisphere).

We observed a significant genotype-by-diagnosis interaction in the volumes of the left pars triangularis (F_(1, 201)_ = 11.074, FDR-corrected P (P_corr_) = 0.027), the supramarginal gyrus (F_(1, 201)_ = 11.832, P_corr_ = 0.027), the superior parietal lobule (F_(1, 201)_ = 14.179, P_corr_ = 0.017), the right posterior midcingulate (F_(1, 201)_ = 9.158, P_corr_ = 0.043), and the frontomarginal gyrus (F_(1, 201)_ = 9.508, P_corr_ = 0.043), as shown in Table 2 and Fig. 1. However, we could not find significant genotype or diagnosis effects in the above-mentioned cortical regions, which had significant diagnosis-by-genotype interactions (all p > 0.1, Table S3).

**Figure 2:**
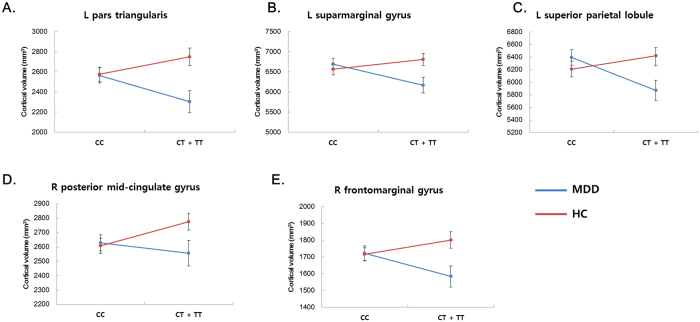
Interaction plot of the cortical volumes with significant diagnosis-by-genotype interactions. (Panel A = left pars triangularis, panel B = left supramarginal gyrus, panel C = left superior parietal lobule, panel D = right posterior midcingulate gyrus, panel E = right frontomarginal gyrus; MDD, major depressive disorder; HC, healthy controls; CC, CC genotype of rs1360780; CT + TT, CT or TT genotype of rs1360780; L, left hemisphere; R, right hemisphere; error bars represent the standard error of the mean).

In the post-hoc analysis of gray matter volumes with significant diagnosis-by-genotype interactions after FDR correction (P_corr_ < 0.05), we found that T allele carriers (TT or CT genotype) had smaller volumes in the left pars triangularis (F_(1, 113)_ = 9.353, uncorrected P (P_uncorr_) = 0.003), the supramarginal gyrus (F_(1, 113)_ = 11.172, P_uncorr_ = 0.001), the superior parietal lobule (F_(1, 113)_ = 14.819, P_uncorr_ < 0.001), and the right frontomarginal gyrus (F_(1, 113)_ = 9.815, P_uncorr_ = 0.002) compared to C allele homozygotes in the MDD group, while no significant differences between genotypes were observed in the healthy control group (Fig. 2 and Table S4). In contrast to the above findings, T allele carriers had an increased volume in the right posterior midcingulate gyrus (F_(1, 87)_ = 5.113, P_uncorr_ = 0.026) compared to C allele homozygotes in the healthy control group, while no significant differences were observed in the MDD group (Fig. 2 and Table S4). Post-hoc analyses of cortical volume and thickness in regions with significant diagnosis-by-genotype interactions without FDR corrections (P_uncorr_ < 0.05) are shown in Tables S5–S7.

We observed a significant genotype effect on the volume of the right subcentral gyrus. In all participants in both the MDD and the healthy control group, T allele carriers had smaller volumes (F_(1, 201)_ = 19.996, P_corr_ = 0.001) compared to C allele homozygotes (Table 2 and Fig. 1). However, we could not find a significant genotype effect on cortical thickness or subcortical volume (Table S3).

Patients with MDD had a trend for a thinner cortex in the right anterior cingulate gyrus (F_(1, 201)_ = 11.909, P_corr_ = 0.052; Table S3). We found no significant diagnosis effect in our cortical volume analysis (Table S3). We also could not find any significant effects for diagnosis, genotype, or their interaction in the analysis of subcortical volume, as shown in Table S3.

### The DNA methylation of the *FKBP5* gene and gray-matter volumes and cortical thickness

The DNA-methylation percentage of the two CpGs did not differ between the diagnostic groups (MDD vs. healthy controls) or genotype groups (CC vs. CT + TT). There was no significant diagnosis-by-genotype interaction for the DNA-methylation percentage of the two CpGs. We observed that the T allele was associated with a reduced methylation of the CpG in pos 1 (F = 4.812, P = 0.032) only in the MDD group. The details are described in Tables S8 and S9.

**Figure 3:**
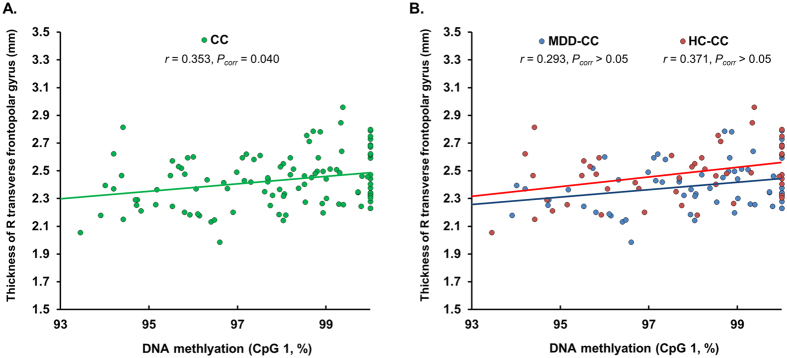
The graphs indicate a significant positive correlation between the percentage of DNA methylation in CpG 1 in intron 7 of the *FKBP5* gene and cortical thickness. Panel A indicates the positive correlation between methylation status and cortical thickness of the right transverse frontopolar gyrus in C homozygote individuals (r = 0.353, P_corr_ = 0.040). Panel B represents the post-hoc analysis of the correlations in each diagnostic group within the C homozygote group (C homozygous MDD patient group: r = 0.293, P_corr_ > 0.05; C homozygous HC group: r = 0.371, P_corr_ > 0.05). Trend lines (green, C homozygote group; blue, C homozygous MDD patient group; red, C homozygous HC group) obtained from bivariate linear regression analyses are presented in the graphs. The Correlation analysis was performed using a two-tailed Pearson’s partial correlation adjusted for age, gender, education level, medication, diagnosis, and total intracranial cavity volume. No significant correlations were observed in the other brain regions or subgroups. (MDD, major depressive disorder; HC, healthy control; CC, C allele homozygous individuals; MDD-CC. C allele homozygous patients with MDD; HC-CC, C allele homozygous healthy controls).

Significant correlations between methylation and volume or cortical thickness are summarized in Table 3 and Fig. 3. In a combined sample of the MDD patient group and healthy control group, C homozygous individuals showed a significant positive correlation between CpG methylation in pos 1 and thickness of the right transverse frontopolar gyrus (r = 0.353, P_corr_ = 0.040), and the covariate effect of diagnosis was significant in this correlation (P = 0.002). In the post-hoc analysis, we analyzed the correlations between CpG methylation in pos 1 and right transverse frontopolar gyrus thickness in each diagnostic group within the C homozygote group, and observed positive correlations in each diagnostic group at a non-significant level (C homozygous MDD patient group: r = 0.293, P_uncorr_ = 0.023, P_corr_ > 0.05; C homozygous healthy control group: r = 0.371, P_uncorr_ = 0.009, P_corr_ > 0.05, Fig. 3). We also observed that DNA methylation did not show any interactions with diagnosis, genotype, and both diagnosis and genotype in terms of the correlations with structural MRI measures (all, P_corr_ > 0.05). The details are described in Tables S10–S12.

## Discussion

Our investigation firstly demonstrated that *FKBP5* rs1360780 genotype and MDD diagnosis have significant interactive effects on gray-matter volumes of several brain regions including the pars triangularis, frontomarginal gyrus, supramarginal gyrus, superior parietal lobule, and posterior midcingulate gyrus. We also found an allele-specific positive correlation of the *FKBP5* gene DNA methylation with thickness of the transverse frontopolar gyrus.

Structural alterations of the above-mentioned cortical regions have been reported in previous imaging studies on MDD. Gray matter volume reduction in the ventrolateral prefrontal cortex (VLPFC), which includes the pars triangularis of the inferior frontal gyrus, was reported in a pooled meta-analysis of 14 neuroimaging studies in medication-naïve patients with MDD^28^. The frontomarginal gyrus is a part of the OFC^29^,^30^. Volume reductions of the OFC in MDD are also reported by several meta-analyses of volumetric studies in patients with MDD^31^,^32^. The posterior midcingulate and dorsal posterior cingulate gyri are adjacent subdivisions of the cingulate cortex and share similar functions^33^,^34^. Cortical thinning or gray matter volume changes in these two cortical regions have been reported in patients with MDD^35^,^36^,^37^,^38^. Reduced cortical volume^39^ and cortical thinning^40^ of the supramarginal gyrus has been reported in patients with MDD in previous studies. In addition, reduced cortical gray matter volume of the superior parietal region has been reported in patients with MDD^41^.

We observed a trend for a thinner cortex in the anterior cingulate gyrus, which is consistent with results of a recent meta-analysis by Schmaal *et al*.^27^, who reported that adult patients with MDD have thinner cortices in the anterior and posterior cingulate cortex and the medial and lateral OFC compared to healthy controls. We also found thinner cortices in the straight, orbital, and ventral posterior cingulate gyri, but only when using uncorrected statistics (Table S3). However, gray matter volumes with significant diagnosis-by-genotype interactions, including the superior parietal lobule, the supramarginal gyrus, the posterior midcingulate gyrus, the frontomarginal gyrus, and the pars triangularis did not have significant volume changes as an effect of diagnosis or genotype. Our negative findings regarding the effects of MDD on cortical volume are inconsistent with the results of a recent meta-analysis on voxel-based morphometry with a large sample, which reported decreased cortical volumes in the insula, the ventromedial prefrontal cortex, and the anterior and posterior cingulate cortices in patients with MDD patients^42^. We believe that this may weaken our conclusion that there is an association between brain structural changes and *FKBP5* genotype.

In the dysfunctional cortico-limbic network model of depression, top-down inhibitory regulation of negative emotion originating in the amygdala, the ventral striatum, and the thalamus is carried out by the lateral part of the prefrontal cortex (PFC), which includes the dorsolateral prefrontal cortex (DLPFC) and the VLPFC, through cognitive and voluntary emotional control^1^,^43^. In addition, the medial part of the PFC, which includes the OFC, the ACC, and the ventromedial PFC, is involved in this process through automatic and implicit emotional control^1^,^43^. These voluntary and automatic regulatory sub-processes of emotion are carried out simultaneously in the reappraisal of emotional context and the generation of emotion^43^. Thus, dysfunction in the normal top-down regulation of these two sub-processes induces overwhelming feelings of negative emotion and eventual depressive mood^43^. The pars triangularis, which is involved in speech production and is activated during semantic processing^44^, is a sub-region of the VLPFC. Abnormal activity of the VLPFC during the cognitive control of negative emotional stimuli or during affective tasks, has been reported in youths^45^ and adults with MDD^43^. Abnormal resting-state activity of the frontomarginal gyrus has been suggested by fMRI studies on MDD^46^,^47^. In addition, the posterior midcingulate and dorsal posterior cingulate gyri are involved in the modulation of negative affect^33^, the emotional processing of aversive social stimuli^48^, and emotional regulation via cognitive reappraisal^49^. Our observation of significant interactive effects between *FKBP5* genotype and MDD diagnosis in the above-mentioned cortical regions indicates that the *FKBP5* gene may be associated with structural changes of neural circuits related to emotional control and mood regulation in MDD, and is relevant to our *a priori* hypothesis.

The study by Tozzi *et al*.^23^, using fMRI data and diffusion tensor images (DTIs), supports our observation of significant interactive effects on pars triangularis, frontomarginal gyrus, superior parietal lobule, and posterior midcingulate gyrus. They showed that in their fMRI analysis, rs1360780 and the diagnosis of MDD had significant interactive effects on OFC and superior parietal lobule, and MDD patients with T allele demonstrated decreased activity during geometrical trials in pars triangularis, and during an emotional task in the posterior cingulate cortex compared with homozygous C patients. They also observed that in their analysis on DTIs, the mean diffusivity of pars triangularis is increased in the patients with T allele compared with homozygous C patients, which reflects axonal degeneration and demyelination in that region. Another study by Fujii *et al*.^24^, investigating the association of rs1360780 with gray-matter volume change in a non-clinical Japanese population, found that T allele carriers had a significant volume reduction in the left posterior cingulate region compared to non-T allele carriers. Even though the result of Fujii *et al*. is limited to a non-clinical population, we guardedly think that this too could support our observation of genotype-by-diagnosis interaction and T-allele associated volume reduction in the posterior midcingulate gyrus.

Considering the previously reported association of heightened HPA-axis activity and hypercortisolemia with gray-matter volume reduction or thinning of several cortical regions, including PFC, OFC, and ACC in MDD^19^,^20^,^50^, the interactive effects of genotype and diagnosis on structural brain changes in our study are congruent with the suggestion that the T allele is associated with a prolonged increase in cortisol levels and impaired negative feedback on the HPA axis^12^,^17^. However, measurements of HPA-axis activity are lacking in our study, and the mediating effect of disturbed HPA-axis function on structural brain changes in MDD patient with T allele might be presumptive. Future studies are required to elucidate the neurobiological mechanisms underlying this issue.

Allele-specific epigenetic modifications of intron 7 of the *FKBP5* gene have been suggested by Klengel *et al*.^16^. We however could not find any significant diagnosis-by-genotype interactions or genotype effects on the percentage of DNA methylation. This negative finding may weaken our conclusion that epigenetic changes to *FKBP5* are correlated with brain structural changes according to genotype or the diagnosis of MDD. In our exploratory analysis, we found that only the T allele is associated with reduced methylation of one CpG locus and only in the MDD group. We observed no significant differences in methylation according to genotype in the healthy control group. This finding may be consistent with reports by Klengel *et al*. that interactive effects between the T allele and childhood trauma are associated with reduced *FKBP5* DNA methylation^16^. However, this study focused on traumatized individuals rather than patients with MDD, thus, we can only postulate from the results of this study, the association between the T allele and reduced methylation in MDD. Another study on this issue by Höhne *et al*. suggested that remitted patients with MDD showed a non-significant trend of increased methylation compared to healthy controls only in the T allele homozygote group other than the CC or CT groups^25^, and this finding is inconsistent with our observation. We cannot provide the exact neurobiological background of this difference, however, we suspect that the discrepancy in sample size (MDD patients, n = 61; healthy controls, n = 55 in Höhne *et al*.^25^) may have had influence on such difference. We believe that further studies are required to elucidate the associations among *FKBP5* genotype, methylation and MDD, as there is limited evidence on this issue. According to previous suggestions^12^,^16^, T-allele-specific reduced methylation is associated with the development of psychiatric disorders. Considering the numerous reports on thinning of several brain regions related to the cortico-limbic network in MDD^27^, a positive correlation between cortical thickness and DNA methylation in the MDD patient group or T-allele carrier group could be expected. However we observed a positive correlation only in the C homozygote group. We could not clearly explain why a positive correlation between methylation and cortical thickness of the right transverse frontopolar gyrus was present in C allele homozygous participants rather than in T-allele carriers or patients with MDD. This highlights the need for further studies to elucidate the exact neurobiological background of our observations.

In this study, we determined both cortical volume and thickness as intermediate phenotypes of the *FKBP5* gene, based on the cumulative evidences that these two brain structural parameters could be influenced by various genetic candidates involved in the pathophysiology of MDD^3^. Both alterations in cortical volume and thickness reflect brain morphologic changes related with MDD^27^,^42^, and there have even been several neuroimaging studies simultaneously investigating changes in both parameters in patients with MDD^51^,^52^. As cortical volume is influenced both by cortical thickness and surface area^53^, we followed up our positive results on cortical volume by exploring the effects on (regional) cortical surface area, again using the Destrieux atlas as implemented in FreeSurfer software and using the same statistical models. After multiple test correction, we found no significant results in both the genotype and methylation related analyses (Tables S13 and S14). Yet, we observed that in 5 of 6 regions for which significant diagnosis-by-genotype interactions or genotype effects on gray-matter volume had been detected, there were at least nominally effects on surface area (Table S13). We suspect that our volumetric abnormalities in interaction of *FKBP5* genotype and MDD might be attributed to a combination of cortical thickness and area abnormalities.

To our knowledge, our study is the first to investigate the associations of *FKBP5* rs1360780 variants and DNA methylation with cortical and subcortical gray-matter volumes and cortical thickness with regard to MDD. The sample size of this study was relatively large compared to recent genetic imaging studies of the *FKBP5* gene^18^,^23^,^54^,^55^. Although our study has multiple strengths, there are several limitations to consider. First, approximately 53.5% of our MDD patients were taking antidepressants, and several studies have reported relationships between brain structural changes and antidepressant treatments^56^,^57^,^58^. However, we have adjusted for antidepressant use by including it as a covariate in all statistical analyses. There was no significant difference in the proportion of genotype subgroups (CC vs. T allele carrier) between medicated and drug-naïve patients with MDD (Table 1), and antidepressant treatment did not influence methylation of the *FKBP5* gene in our study (Table S15). Second, we have postulated that the influence of *FKBP5* genetic variants and epigenetic modifications on structural brain changes is mediated by disturbances in the HPA axis and its influence on the brain. However, we did not investigate HPA-axis activity in our participants by measuring plasma cortisol or adrenocorticotropic hormone (ACTH). Thus, we could not elucidate a clear causal relationship between these genetic or epigenetic markers and morphological brain changes in MDD. Third, even though there has been an emphasis on the interactions between *FKBP5* genetic variant, epigenetic changes, and childhood trauma^15^, we did not assess childhood trauma in the participants. With regard to volume reductions in MDD patients with the T allele, we could not clearly differentiate whether rs1360780 interacted with diagnosis-specific effects or childhood trauma, considering the high prevalence of childhood trauma in MDD patients. We also could not determine the mediating or moderating effects of childhood adversity on the relationship between DNA methylation status and structural brain alterations from our findings. Finally, we could not find an association between genotypic distribution of rs1360780 and predisposition to MDD. However, this study demonstrated a significant association between the risk allele and structural alterations in neural networks of emotion processing in patients with MDD. Furthermore, previous imaging genetic studies on *FKBP5* rs1360780 also did not detect any differences in genotypic distribution between MDD and healthy control groups^23^. Our findings may provide evidence that allelic variants of rs1360780 could influence brain morphologic changes related to the pathophysiology of MDD.

In summary, we have demonstrated that the *FKBP5* rs1360780 genotype and MDD have interactive effects on gray-matter volumes of several cortical regions involved in emotion processing and mood regulation, and that epigenetic changes to *FKBP5* are correlated with cortical thickness according to *FKBP5* genotype. We hope that our findings will provide additional neurobiological evidence for the influence of the *FKBP5* gene on structural brain changes in patients with MDD.

## Method

### Participants

A total of one hundred and fourteen patients diagnosed with MDD were recruited from the outpatient psychiatric clinic of Korea University Anam Hospital, located in Seoul, Republic of Korea. We included adults diagnosed with MDD, aged 20–69 years. The diagnosis of MDD was made by a board-certified psychiatrist based on the DSM-IV criteria, and confirmed by an independent psychiatrist using the Structured Clinical Interview for DSM-IV Axis I disorders (SCID-I). Their concordance for the diagnosis of MDD was 0.95. The exclusion criteria were as follows: (1) presumptive primary comorbid diagnosis of any other major psychiatric illness (based on DSM-IV criteria) on Axis I or Axis II, within the last 6 months; (2) MDD with psychotic features; (3) serious or unstable medical illness; (4) primary neurological illness, and (5) any contraindication for MRI. We assessed the duration of illness for MDD in an interview using the life-chart methodology. Eighty-eight healthy participants without histories of any psychiatric diagnoses were recruited as the control group through advertisements from the community. All participants in both groups were right-handed, according to the Edinburgh Handedness Test^59^. The severity of depressive symptoms of the participants in both groups was evaluated on the same day as the MRI scans, using HDRS^60^. At study enrollment, 61 patients with MDD were taking antidepressants, and 53 patients were medication-naïve. The details are described in Table 1. The study protocol was approved by the Institutional Review Board of Korea University Anam Hospital in accordance with the Declaration of Helsinki (revised in 2008), and all participants gave informed consent to participate in the study.

### Genotyping and Methylation analysis

The *FKBP5* rs1360780 was genotyped using genomic DNA extracted from peripheral venous blood of each participant, and analyzed according to a previously described standard protocol^61^. Polymerase chain reactions were performed using the following primers: forward, 5′-GCCAAATTCCAGGCAAAGGG-3′; and reverse, 5′-GATCAGCGGATGGTGGGAGG-3′. The genotyping success rate was above 95%. The allele frequencies (C allele/T allele) were as follows: patients with MDD 0.80/0.20, healthy controls 0.82/0.18. We divided the genotype groups based on the recessive model (comparing carriers of the risk-allele with non-risk allele homozygotes) in accordance with previous imaging studies on the *FKBP5* rs1360780 18,23,24. The genotype distribution and Hardy-Weinberg equilibrium of the two groups were assessed using a chi-square test (Table 1).

We performed pyrosequencing of bisulfite-treated genomic DNA for the methylation analysis of intron 7 of the *FKBP5* gene. Among the intronic regions of *FKBP5* gene, intron 7 has been most thoroughly investigated regarding the association of DNA methylation with transcriptional activation of *FKBP5*, childhood trauma, and predisposition to psychiatric disorders^14^,^16^,^26^, thus we selected intron 7 as our target for the DNA methylation analysis. The details of the sodium bisulfite treatment and pyrosequencing method and the primer sequence are described in the supplement. Among a total of 202 participants, we performed a methylation analysis in 186 participants, as we failed to analyze the DNA methylation status of 10 MDD patients and 6 healthy controls during the bisulfite pyrosequencing process. We confirmed that there were no biases on demographic characteristics or genotypic distribution of the sample included in the methylation analysis. The detailed data on the sample are described in Table S1. The methylation percentage was calculated by averaging the degree of methylation at two CpG sites detected by pyrosequencing.

### MRI data acquisition

MRI scans were acquired parallel to the anterior-commissure–posterior-commissure line using a 3.0 T Siemens Trio whole-body imaging system (Siemens Medical Systems, Iselin, NJ, USA), using 3D T1-weighted magnetization-prepared rapid gradient-echo (MP-RAGE) with the following parameters: 1900 ms repetition time, 2.6 ms echo time, 220 mm field of view, 256 × 256 matrix size, 1 mm slice thickness, 176 coronal slices without gap, 0.86 × 0.86 × 1 mm^3^ voxels, 16° flip angle, number of excitations = 1. After individual’s MRI scanning, the artifacts of MRI system and motion were visually checked, and when the artifacts were observed, the subject’s MRI was rescanned.

### Image processing

The cortical and subcortical gray-matter volumes and cortical thickness were calculated using the automated procedure for volumetric measures of whole brain structures implemented in the FreeSurfer 5.3 development version (Massachusetts General Hospital, Boston, U.S., http://surfer.nmr.mgh.harvard.edu) from T1 imaging. We used the demeaned value for the total intracranial cavity volume (TICV) measured manually^62^ to normalize the regional brain volume.

A three-dimensional model of cortical surface reconstructions computed from T1 images was used in the FreeSurfer. Details of the technical aspects of these procedures have been described in previous publications^63^,^64^,^65^. In summary, removal of non-brain tissue, automated Talairach transformation of each subject’s native brain, segmentation of the volumetric structures^64^,^66^, inflation of the cortical surface to an average spherical surface, intensity normalization, and automated topology correction were performed^67^,^68^. The transition between gray/white matter and the pial boundary were determined by detecting the greatest shift in intensity through surface deformation. The entire cortex of each subject was then visually inspected, and data from subjects with inaccuracies in segmentation were discarded. Each hemisphere was then automatically parcellated into 74 distinct cortical regions consisting of gyri and sulci by a previously described method^69^, and the volume and thickness of these cortical regions were automatically calculated. From the 74 cortical gyri and sulci, we used cortical volume and thickness data for 38 cortical gyri in the analysis of cortical volume and thickness (Table S2 and Figure S1). The subcortical volumes were also calculated using the automated segmentation algorithm, assignment of a neuroanatomical label to each voxel, and volumetric measurement procedures implemented in the FreeSurfer. We obtained seven subcortical (thalamus, caudate nucleus, putamen, globus pallidum, hippocampus, amygdala, and nucleus accumbens) gray-matter volumes.

### Statistical Analyses

We performed a comparison of cortical and subcortical gray-matter volumes and cortical thicknesses in the two diagnostic groups (patients with MDD vs. healthy controls) and in the *FKBP5* rs1360780 genotype groups (CC vs. CT + TT), and further investigated diagnosis-by-genotype interactions in our main analysis. The diagnosis-by-genotype interaction was determined to be the main outcome of our study. Automatically calculated data for the cortical and subcortical gray-matter volumes, and cortical thickness values obtained using FreeSurfer were analyzed using a two-way analysis of covariance (ANCOVA), with individual volumes and thickness as dependent variables; diagnosis and genotype as independent variables; and age, gender, education level, and medication (entered as medication status: 0 for medication-naïve patients with MDD and healthy controls, and 1 for patients with MDD on antidepressant treatment), and TICV as covariates. After the main analysis, we investigated the effects of genotype on gray matter volume or thickness in regions with significant diagnosis-by-genotype interactions within each diagnosis group (MDD and healthy control group) as a post-hoc analysis. We evaluated differences in the DNA methylation status of the two CpGs in intron 7 of the *FKBP5* gene between the diagnostic and genotype groups using an ANCOVA adjusted for age, gender, and medication. In order to investigate the correlations between the percentage of DNA methylation in intron 7 of this gene and cortical and subcortical gray-matter volumes and cortical thickness according to diagnosis and genotype, a two-tailed Pearson’s partial correlation was performed separately for each group determined by diagnosis (MDD patients and healthy controls) and genotype (T allele carriers and C allele homozygotes). In the Pearson’s partial correlation analysis between methylation and brain structural outcomes, age, gender, education level, medication, and TICV were adjusted as covariates. Specifically, in the correlation analysis regarding diagnostic groups, genotype (T allele carriers vs. C allele homozygotes) was included as an additional covariate to control for genotypic effects, while in the correlation analysis regarding genotype groups, diagnosis (MDD patients vs. healthy controls) was included as an additional covariate to control for diagnostic effects. We also investigated the interaction of DNA methylation with diagnosis, genotype, or both diagnosis and genotype in terms of the correlations with structural MRI measures using the hierarchical moderated regression analysis which has been used in previous psychiatric genetic studies^70^,^71^,^72^. In the regression analysis, the same covariates as those used in the main analysis were included. False Discovery Rate (FDR) correction, as described by Benjamini and Hochberg^73^, was applied to each main analysis and the methylation analysis for multiple comparisons correction (q < 0.05). The numbers of comparisons in the main analyses are as follows: cortical volume: 76 comparisons (=38 cortical regions × 2 hemispheres), cortical thickness: 76 comparisons (=38 cortical regions × 2 hemispheres); subcortical volume: 14 comparisons (=7 subcortical regions × 2 hemispheres). The numbers of comparisons in the methylation analyses are as follows: for cortical gray-matter volumes or thickness in the analysis according to diagnostic groups: 304 comparisons (=38 cortical regions × 2 hemispheres × 2 diagnostic groups × 2 CpG sites); genotype groups: 304 comparisons (=38 cortical regions × 2 hemispheres × 2 genotype groups × 2 CpG sites); and interactive effects (e.g. diagnosis-by-methylation, genotype-by-methylation, diagnosis-by-genotype-by-methylation): 456 comparisons (=38 cortical regions × 2 hemispheres × 3 interactions × 2 CpG sites); for subcortical volumes in the analysis according to diagnostic groups: 56 comparisons (=7 subcortical regions × 2 hemispheres × 2 diagnostic groups × 2 CpG sites); genotype groups: 56 comparisons (=7 subcortical regions × 2 hemispheres × 2 genotype groups × 2 CpG sites); and interactive effects: 84 comparisons (=7 subcortical regions × 2 hemispheres × 3 interactions × 2 CpG sites). FDR correction was applied to each analysis of diagnostic effect, genotypic effect, and interaction effects and each brain structural outcome (cortical and subcortical gray-matter volumes and cortical thickness) separately in the main and methylation analyses. To analyze group differences due to demographic and clinical characteristics, age and HDRS scores were analyzed using t-tests, and the distributions of gender, education level, and drug-naïve status based on genotype were analyzed using chi-squared tests. Statistical analyses were performed using SPSS version 18.0 (SPSS Inc., Chicago, IL, USA).

## Additional Information

**How to cite this article**: Han, K.-M. *et al*. Influence of *FKBP5* polymorphism and DNA methylation on structural changes of the brain in major depressive disorder. *Sci. Rep.* **7**, 42621; doi: 10.1038/srep42621 (2017).

**Publisher's note:** Springer Nature remains neutral with regard to jurisdictional claims in published maps and institutional affiliations.

## Supplementary Material

Supplementary Information

## Figures and Tables

**Table 1:** Demographic and clinical characteristics of patients with major depressive disorder and healthy controls.

	MDD (n = 114)	HC (n = 88)	p value
Age	43.51 ± 12.0	39.89 ± 14.05	0.055
Gender (female)	90	61	0.142
Education level			
Elementary and middle school	27	13	0.117
High school or college/university	80	64	
Above graduate school	7	11	
HDRS-17 score	14.81 ± 8.02	2.27 ± 2.10	<0.001
Duration of illness (months)	45.18 ± 47.47		
*FKBP5* gene rs1360780			
CC	72	58	0.725
CT	39	29	
TT	3	1	
HWE	0.394	0.204	
CC	72	58	0.767
CT + TT	42	30	
Drug-naïve/Antidepressant	53/61		
CC	32/40		0.697
CT + TT	21/21		
Antidepressant type			
SSRI	30		
SNRI	10		
NDRI	6		
NaSSA	4		
Combination	11		

Data represent mean ± standard deviation for age, HDRS-17 scores, and duration of illness. The p values for distributions of gender, education level, *FKBP5* genotype, and drug-naïve patients according to the genotype were obtained by chi-square test. The p values for comparisons of age and HDRS-17 scores were obtained by independent t-test. Allele frequencies (C/T): MDD patients 0.80/0.20, HC subjects 0.82/0.18. MDD, major depressive disorder; HC, healthy controls; HDRS-17, Hamilton Depression Rating Scale; HWE, Hardy-Weinberg equilibrium; SSRI, selective serotonin reuptake inhibitor; SNRI, serotonin and norepinephrine reuptake inhibitor; NDRI, norepinephrine-dopamine reuptake inhibitor; NaSSA, noradrenergic and specific serotonergic antidepressant; Combination, combinations of two or more types of antidepressant.

**Table 2:** Summary of differences in volume and thickness of various brain regions among groups determined by genotype and diagnosis.

Brain regions	MDD (n = 114) vs. HC (n = 88)			CC (n = 130) vs. CT + TT (n = 72)				Diagnosis × Genotype interaction		
	F	P_uncorr_	P_corr_	F	P_uncorr_	P_corr_		F	P_uncorr_	P_corr_
***cortical volume***										
L pars triangularis	1.387	>0.1	>0.1	1.223	>0.1	>0.1		11.074	0.001*	0.027*
L supramarginal gyrus	0.059			2.391				11.832	0.001*	0.027*
L superior parietal lobule	0.896			2.704				14.179	<0.001*	0.017*
R posterior mid-cingulate gyrus	0.089			0.027				9.158	0.003*	0.043*
R frontomarginal gyrus	1.337			0.935				9.508	0.002*	0.043*
R subcentral gyrus	0.367			19.996	<0.001*	0.001*	CC > CT + TT	1.395	0.239	>0.1

The F and uncorrected P values were obtained using analysis of covariance (ANCOVA) adjusted for age, gender, education level, medication, and total intracranial volume as covariates. Only brain regions with significant difference or interaction after the multiple comparison correction are shown. The False Discovery Rate (FDR) was applied in each analysis for multiple comparison correction, q < 0.05; cortical volume: 76 comparisons in both hemispheres; cortical thickness: 76 comparisons in both hemispheres; subcortical volume: 14 comparisons in both hemispheres. ^*^Regions that remained significant after the multiple comparison correction are marked with an asterisk. MDD, major depressive disorder; HC, healthy controls; CC, CC genotype of rs1360780; CT + TT, CT or TT genotype of rs1360780; L, left hemisphere; R, right hemisphere; P_uncorr,_ uncorrected P-value; P_corr_, FDR-corrected P-value.

**Table 3:** Summary of correlation analyses among gray-matter volumes or cortical thickness and DNA methylation in intron 7 of the *FKBP5* gene among groups determined by diagnosis and genotype.

Subgroups	Brain regions	r	P_uncorr_	P_corr_	CpG
***cortical thickness***					
CC (n = 118)	R transverse frontopolar gyrus	0.353	<0.001	0.040	pos 1

A two-tailed Pearson’s partial correlation was performed to analyze the correlations of *FKBP5* gene DNA methylation with cortical gray-matter volumes or thickness adjusting for age, gender, education level, medication, total intracranial cavity volume, *FKBP5* genotype (only included in the analyses of MDD or HC groups), and diagnosis (only included in the analyses of T allele carrier or C homozygote groups). A hierarchical moderated regression analysis was performed to investigated the interactive effect of the DNA methylation with the diagnosis, genotype, or both diagnosis and genotype in terms of the correlation with the cortical gray-matter volumes or thickness. Only regions with significant correlations after multiple comparison correction are shown. The False Discovery Rate (FDR) was applied in each analysis for multiple comparison correction, q < 0.05; for cortical gray-matter volumes or thickness in the analysis of diagnostic effect (MDD or HC group): 304 comparisons (=38 cortical regions × 2 hemispheres × 2 diagnostic groups × 2 CpG sites); genotype effect (T allele carrier or C homozygote group): 304 comparisons (=38 cortical regions × 2 hemispheres × 2 genotype groups × 2 CpG sites); and interactive effects: 456 comparisons (=38 cortical regions × 2 hemispheres × 3 interactions × 2 CpG sites); for subcortical volumes in the analysis of diagnostic effect: 56 comparisons (=7 subcortical regions × 2 hemispheres × 2 diagnostic groups × 2 CpG sites); genotype effect: 56 comparisons (=7 subcortical regions × 2 hemispheres × 2 genotype groups × 2 CpG sites); and interactive effects: 84 comparisons (=7 subcortical regions × 2 hemispheres × 3 interactions × 2 CpG sites). MDD, major depressive disorder; HC, healthy control; CC, participants with CC genotype of rs1360780; R, right hemisphere; P_uncorr,_ uncorrected P value; P_corr_, FDR-corrected P-value.
