# Supplementary material for: Influence of FKBP5 polymorphism and DNA methylation on structural changes of the brain in major depressive disorder
Source: Sci Rep. 2017 Feb 15;7:42621. doi: 10.1038/srep42621 (PMC5309810; doi:10.1038/srep42621)
Supplement: Supplementary Information [file srep42621-s1.pdf]

Influence of *FKBP5* polymorphism and DNA methylation on structural changes of the brain in major depressive disorder

**- Supplementary information -**

Kyu-Man Han MD[a*], Eunsoo Won, MD, PhD[a*], Youngbo Sim, BA[b], June Kang, MA[c], Changsu Han, MD, PhD[d], Yong-Ku Kim, MD, PhD[d], Seung-Hyun Kim, MD[e], PhD, Sook-Haeng Joe, MD, PhD[e], Min-Soo Lee, MD, PhD[a], Woo-Suk Tae, PhD[b†], Byung-Joo Ham MD, PhD[a,b†]

[a]Department of Psychiatry, Korea University Anam Hospital, Korea University College of Medicine, Seoul, Republic of Korea; [b]Brain Convergence Research Center, Korea University Anam Hospital, Seoul, Republic of Korea; [c]Department of Biomedical Sciences, Korea University College of Medicine, Seoul, Republic of Korea; [d]Department of Psychiatry, Korea University Ansan Hospital, Korea University College of Medicine, Seoul, Republic of Korea; [e]Department of Psychiatry, Korea University Guro Hospital, Korea University College of Medicine, Seoul, Republic of Korea.

*The first two authors contributed equally to this article.

[†]Corresponding author at: Department of Psychiatry, Korea University Anam Hospital, 73, Inchon-ro, Seongbuk-gu, Seoul 02841, Republic of Korea. Tel +82-2-920-6843, Fax +82-2-927-2836, *E-mail address:* hambj@korea.ac.kr (B.-J. Ham); Brain Convergence Research Center, Korea University Anam Hospital, 73, Inchon-ro, Seongbuk-gu, Seoul 02841, Republic of Korea. Tel: +82-2-2286-1057, Fax: +82-2-923-1036 *E-mail address:* wstae@korea.ac.kr (W.-S. Tae).

**The methods for Sodium Bisulfite modification and Pyrosequencing.**

Bisulfite modified gDNA was prepared using the EZ DNA Methylation-Lighting$^{TM}$ kit (Zymo Research, USA) according to the manufacturer's instructions. The bisulfite reaction was carried out on 500 ng gDNA. The reaction volume was adjusted to 20 μL with sterile water and 130 μL of CT conversion Reagent was added. Sample tubes were placed in a thermal cycler (MJ Research) and the following steps were performed: 8 min at 98°C, 60 min at 54°C, and storage at 4°C for up to 20 h.

DNA was purified using the EZ DNA Methylation-Lighting$^{TM}$ kit (Zymo Research, USA). The converted samples were added to a Zymo-Spin IC$^{TM}$ Column containing 600 μL of the M-Binding Buffer and mixed by inverting the column several times. The column was centrifuged at full speed for 30 s and the flow-through was discarded. The column was washed by adding 200 μL of M-Wash Buffer and centrifuging at full speed. Then 200 μL of M-Desulphonation Buffer was added to the column, which was incubated at room temperature (20–30°C) for 15–20 min. After incubation, the column was centrifuged at full speed for 30 s. The column was washed twice by adding 200 μL of M-Wash Buffer and centrifuging at full speed. The converted gDNA was eluted by adding 20 μL of M-Elution Buffer to the column and centrifuging. DNA samples were finally stored at −20°C until further use.

The primers were designed using Pyrosequencing Assay Design Software v2.0 (Qiagen). The PCR reaction was carried out in a volume of 20 μL with 20 ng or more of converted gDNA, the PCR premixture (Enzynomics, Korea), 1 μL of 10 pmol/μL Primer-S, and 1 μL of 10 pmol/μL biotinylated-Primer-As. Amplification was carried out according to the general guidelines suggested for Pyrosequencing: denaturing at 95°C for 10 min, followed by 45 cycles of 95°C for 30 s, at 60°C for 30 s, at 72°C for 30 s and a final extension at 72°C for 5 min. The PCR reaction (2 μL) was confirmed by electrophoresis in a 2% Agarose gel and visualized by ethidium bromide staining. The ssDNA template was prepared from 16–18 μL biotinylated PCR product using streptavidin Sepharose® HP beads (Amersham Biosciences, Sweden) following the PSQ 96 sample preparation guide using multichannel pipettes. Fifteen picomoles of the respective sequencing primer were added for analysis. Sequencing was performed on a PyroMark ID system with the Pyro Gold reagent kit (Qiagen) according to the manufacturer's instructions, without further optimization.

**Target CpG islands and the primers for pyrosequencing.**

| Gene | Primer | | Size (bp) |
|---|---|---|---|
| *FKBP5* Intron 7 | Forward | 5′-TGGGATAATAATTTGGAGTTATAGTGTA-3′ | 187 |
| | Biotinylated-reverse | 5′-ATTCCCTTATTTATTCCTAAACTTATTCTT-3′ | |
| | Sequencing primer | 5′-GGAGTTATAGTGTAGGTTTT-3′ | |

Table S1. Demographic and clinical characteristics of patients with major depressive disorder and healthy controls included in the methylation analysis.

| | **MDD (n=104)** | **HC (n=82)** | p value |
|---|---|---|---|
| Age | 43.76 ± 11.91 | 40.13 ± 14.30 | 0.067 |
| Gender (female) | 83 | 56 | 0.090 |
| Education level | | | |
| Elementary and middle school | 24 | 13 | |
| High school or college/university | 73 | 58 | 0.190 |
| Above graduate school | 7 | 11 | |
| HDRS-17 score | 14.68 ± 8.14 | 2.28 ± 2.14 | <0.001 |
| Duration of illness (months) | 46.19 ± 48.48 | | |
| *FKBP5* gene rs1360780 | | | |
| CC | 65 | 53 | |
| CT | 36 | 28 | 0.731 |
| TT | 3 | 1 | |
| HWE | 0.450 | 0.198 | |
| CC | 65 | 53 | 0.878 |
| CT + TT | 39 | 29 | |
| Drug-naïve / Antidepressant | 49 / 55 | | |
| CC | 29 / 36 | | 0.548 |
| CT + TT | 20 / 19 | | |
| Antidepressant type | | | |
| SSRI | 28 | | |
| SNRI | 9 | | |
| NDRI | 5 | | |
| NaSSA | 3 | | |
| Combination | 10 | | |

Data represent mean ± standard deviation for age, HDRS-17 scores, and duration of illness.

The p values for distributions of gender, education level, FKBP5 genotype, and drug-naïve patients according to the genotype were obtained by chi-square test.

The p values for comparisons of age and HDRS-17 scores were obtained by independent t-test.

Allele frequencies (C/T): MDD patients 0.80/0.20, HC subjects 0.82/0.18.

MDD, major depressive disorder; HC, healthy controls; HDRS-17, Hamilton Depression Rating Scale; HWE, Hardy-Weinberg equilibrium; SSRI, serotonin and norepinephrine reuptake inhibitor; SNRI, serotonin and norepinephrine reuptake inhibitor; NDRI, norepinephrine-dopamine reuptake inhibitor; NaSSA, noradrenergic and specific serotonergic antidepressant; Combination, combinations of two or more types of antidepressant.

Table S2. List of the 38 cortical gyri.

| |
|---|
| Frontomarginal gyrus (of Wernicke) |
| Inferior occipital gyrus |
| Paracentral lobule |
| Subcentral gyrus (central operculum) |
| Transverse frontopolar gyrus |
| Anterior part of the cingulate gyrus |
| Middle-anterior part of the cingulate gyrus |
| Middle-posterior part of the cingulate gyrus |
| Dorsal posterior cingulate gyrus |
| Ventral posterior cingulate gyrus (isthmus of the cingulate gyrus) |
| Cuneus |
| Opercular part of the inferior frontal gyrus (Pars opercularis) |
| Orbital part of the inferior frontal gyrus (Pars orbitalis) |
| Triangular part of the inferior frontal gyrus (Pars triangularis) |
| Middle frontal gyrus |
| Superior frontal gyrus |
| Long insular gyrus |
| Short insular gyrus |
| Middle occipital gyrus |
| Superior occipital gyrus |
| Lateral occipito-temporal gyrus |
| Lingual gyrus |
| Parahippocampal gyrus |
| Orbital gyrus |
| Angular gyrus |
| Supramarginal gyrus |
| Superior parietal lobule |
| Postcentral gyrus |
| Precentral gyrus |
| Precuneus |
| Straight gyrus |
| Subcallosal gyrus |
| Anterior transverse temporal gyrus (of Heschl) |
| Lateral aspect of the superior temporal gyrus |
| Planum polare of the superior temporal gyrus |

| |
|---|
| Planum temporale or temporal plane of the superior temporal gyrus |
| Inferior temporal gyrus |
| Middle temporal gyrus |

Figure S1. Cortical surfaces labeled using Destrieux atlas of a subject's right hemisphere.

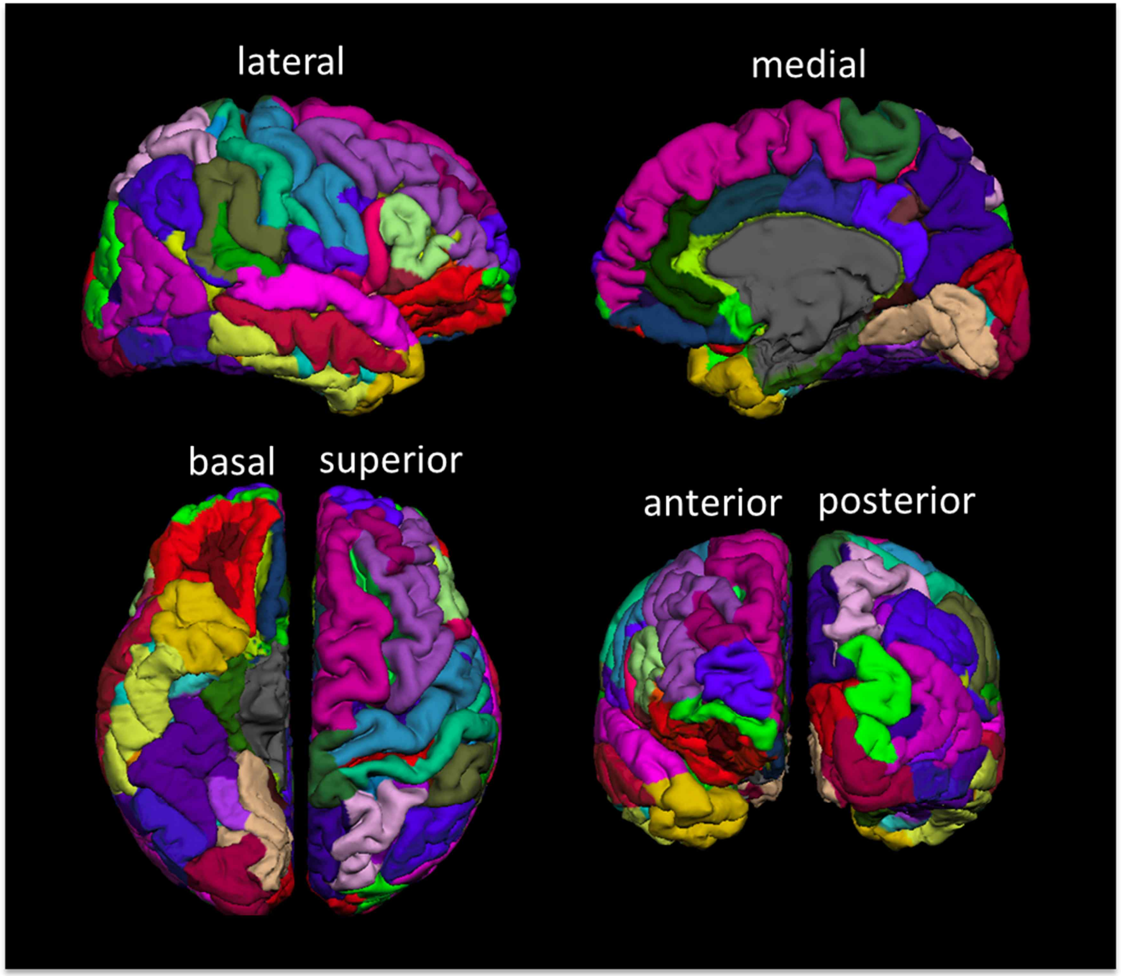

Table S3. Main-analysis investigating effect of diagnosis, genotype, and diagnosis-by-genotype interaction on cortical gray-matter volumes and thicknesses of whole-brain.

| brain regions | MDD vs. HC | | | CC vs. CT + TT | | | | Diagnosis x Genotype interaction | | |
|---|---|---|---|---|---|---|---|---|---|---|
| | F | $P_{uncorr}$ | $P_{corr}$ | F | $P_{uncorr}$ | $P_{corr}$ | | F | $P_{uncorr}$ | $P_{corr}$ |
| ***cortical volume*** | | | | | | | | | | |
| L frontomarginal gyrus | 0.166 | 0.684 | 0.989 | 4.701 | 0.031 | 0.265 | CC > CT + TT | 1.634 | 0.203 | 0.501 |
| L inferior occipital gyrus | 0.145 | 0.704 | 0.989 | 0.335 | 0.563 | 0.669 | | 0.130 | 0.719 | 0.828 |
| L paracentral lobule | 3.049 | 0.082 | 0.782 | 0.429 | 0.513 | 0.639 | | 0.018 | 0.892 | 0.968 |
| L subcentral gyrus | 0.368 | 0.545 | 0.989 | 5.986 | 0.015 | 0.206 | CC > CT + TT | 0.914 | 0.340 | 0.517 |
| L transverse frontopolar gyrus | 3.118 | 0.079 | 0.782 | 1.439 | 0.232 | 0.522 | | 1.239 | 0.267 | 0.501 |
| L anterior cingulate gyrus | 0.96 | 0.328 | 0.989 | 2.324 | 0.129 | 0.409 | | 2.850 | 0.093 | 0.393 |
| L anterior midcingulate gyrus | 0.304 | 0.582 | 0.989 | 0.724 | 0.396 | 0.605 | | 0.422 | 0.517 | 0.689 |
| L posterior midcingulate gyrus | 0.944 | 0.333 | 0.989 | 0.745 | 0.389 | 0.605 | | 3.454 | 0.065 | 0.289 |
| L dorsal posterior cingulate gyrus | 2.177 | 0.142 | 0.989 | 3.672 | 0.057 | 0.281 | | 1.030 | 0.311 | 0.517 |
| L ventral posterior cingulate gyrus | 2.563 | 0.111 | 0.938 | 1.395 | 0.239 | 0.522 | | 1.322 | 0.252 | 0.501 |
| L cuneus | 0.120 | 0.730 | 0.989 | 0.44 | 0.508 | 0.639 | | 0.064 | 0.801 | 0.895 |
| L pars opercularis | 0.004 | 0.952 | 0.989 | 1.166 | 0.282 | 0.522 | | 2.139 | 0.145 | 0.460 |
| L pars orbitalis | 0.002 | 0.965 | 0.989 | 0.402 | 0.527 | 0.645 | | 1.607 | 0.206 | 0.501 |
| L pars triangularis | 1.387 | 0.240 | 0.989 | 1.223 | 0.270 | 0.522 | | 11.074 | 0.001* | 0.027* |
| L middle frontal gyrus | 0.343 | 0.559 | 0.989 | 1.245 | 0.266 | 0.522 | | 1.384 | 0.241 | 0.501 |
| L superior frontal gyrus | 0.026 | 0.873 | 0.989 | 3.593 | 0.060 | 0.281 | | 0.002 | 0.967 | 0.979 |

| | | | | | | | | | | | |
|---|---|---|---|---|---|---|---|---|---|---|---|
| L long insular gyrus | 0.300 | 0.584 | 0.989 | | 5.541 | 0.020 | 0.213 | CC > CT + TT | 0.003 | 0.956 | 0.979 |
| L short insular gyrus | 1.067 | 0.303 | 0.989 | | 0.577 | 0.448 | 0.619 | | 4.897 | 0.028 | 0.152 |
| L middle occipital gyrus | 3.063 | 0.082 | 0.782 | | 0.159 | 0.690 | 0.760 | | 5.545 | 0.020 | 0.124 |
| L superior occipital gyrus | <0.001 | 0.983 | 0.989 | | 0.627 | 0.429 | 0.615 | | 0.452 | 0.502 | 0.689 |
| L lateral occipito-temporal gyrus | 0.087 | 0.769 | 0.989 | | 0.607 | 0.437 | 0.615 | | 0.174 | 0.677 | 0.816 |
| L lingual gyrus | 0.065 | 0.799 | 0.989 | | 0.527 | 0.469 | 0.627 | | 1.347 | 0.247 | 0.501 |
| L parahippocampal gyrus | 0.828 | 0.364 | 0.989 | | 0.523 | 0.471 | 0.627 | | 0.226 | 0.635 | 0.804 |
| L orbital gyrus | 0.65 | 0.421 | 0.989 | | 2.846 | 0.093 | 0.351 | | 1.028 | 0.312 | 0.517 |
| L angular gyrus | 3.304 | 0.071 | 0.782 | | 0.036 | 0.850 | 0.907 | | 0.634 | 0.427 | 0.624 |
| L supramarginal gyrus | 0.059 | 0.809 | 0.989 | | 2.391 | 0.124 | 0.409 | | 11.832 | 0.001* | 0.027* |
| L superior parietal lobule | 0.896 | 0.345 | 0.989 | | 2.704 | 0.102 | 0.351 | | 14.179 | <0.001* | 0.017* |
| L postcentral gyrus | 0.001 | 0.971 | 0.989 | | 5.087 | 0.025 | 0.240 | CC > CT + TT | 0.002 | 0.962 | 0.979 |
| L precentral gyrus | 0.008 | 0.927 | 0.989 | | 0.003 | 0.960 | 0.960 | | 0.517 | 0.473 | 0.678 |
| L precuneus | 0.320 | 0.572 | 0.989 | | 1.174 | 0.280 | 0.522 | | 6.337 | 0.013 | 0.114 |
| L straight gyrus | 0.580 | 0.447 | 0.989 | | 0.219 | 0.641 | 0.727 | | 1.258 | 0.263 | 0.501 |
| L subcallosal gyrus | 5.134 | 0.025 | 0.782 | MDD > HC | 1.519 | 0.219 | 0.522 | | 0.004 | 0.951 | 0.979 |
| L anterior transverse temporal gyrus | <0.001 | 0.989 | 0.989 | | 5.879 | 0.016 | 0.206 | CC > CT + TT | 1.706 | 0.193 | 0.501 |
| L lateral superior temporal gyrus | 0.001 | 0.970 | 0.989 | | 3.289 | 0.071 | 0.301 | | 0.944 | 0.333 | 0.517 |
| L planum polare | 0.241 | 0.624 | 0.989 | | 1.182 | 0.278 | 0.522 | | 1.401 | 0.238 | 0.501 |
| L planum temporale | 0.600 | 0.440 | 0.989 | | 0.717 | 0.398 | 0.605 | | 0.949 | 0.331 | 0.517 |
| L inferior temporal gyrus | 0.982 | 0.323 | 0.989 | | 0.797 | 0.373 | 0.605 | | 1.750 | 0.187 | 0.501 |
| L middle temporal gyrus | 0.153 | 0.696 | 0.989 | | 1.038 | 0.310 | 0.547 | | 1.558 | 0.213 | 0.501 |
| R frontomarginal gyrus | 1.337 | 0.249 | 0.989 | | 0.935 | 0.335 | 0.565 | | 9.508 | 0.002* | 0.043* |
| R inferior occipital gyrus | 0.225 | 0.636 | 0.989 | | 0.448 | 0.504 | 0.639 | | 0.254 | 0.615 | 0.792 |

| | | | | | | | | | | |
|---|---|---|---|---|---|---|---|---|---|---|
| R paracentral lobule | 1.692 | 0.195 | 0.989 | 0.029 | 0.865 | 0.907 | | 1.177 | 0.279 | 0.501 |
| R subcentral gyrus | 0.367 | 0.546 | 0.989 | 19.996 | <0.001* | 0.001* | CC > CT + TT | 1.395 | 0.239 | 0.501 |
| R transverse frontopolar gyrus | 0.053 | 0.818 | 0.989 | 0.631 | 0.428 | 0.615 | | 5.111 | 0.025 | 0.146 |
| R anterior cingulate gyrus | 0.010 | 0.922 | 0.989 | 1.757 | 0.187 | 0.522 | | 1.157 | 0.283 | 0.501 |
| R anterior midcingulate gyrus | 0.564 | 0.454 | 0.989 | 3.647 | 0.058 | 0.281 | | 0.002 | 0.963 | 0.979 |
| R posterior midcingulate gyrus | 0.089 | 0.765 | 0.989 | 0.027 | 0.871 | 0.907 | | 9.158 | 0.003* | 0.043* |
| R dorsal posterior cingulate gyrus | 0.551 | 0.459 | 0.989 | 0.317 | 0.574 | 0.671 | | 6.213 | 0.014 | 0.114 |
| R ventral posterior cingulate gyrus | 0.043 | 0.835 | 0.989 | 1.123 | 0.290 | 0.526 | | 0.915 | 0.340 | 0.517 |
| R cuneus | 0.126 | 0.723 | 0.989 | 1.249 | 0.265 | 0.522 | | 0.373 | 0.542 | 0.710 |
| R pars opercularis | 0.206 | 0.651 | 0.989 | 5.956 | 0.016 | 0.206 | CC > CT + TT | 0.153 | 0.696 | 0.816 |
| R pars orbitalis | 0.016 | 0.898 | 0.989 | 0.788 | 0.376 | 0.605 | | 2.013 | 0.158 | 0.479 |
| R pars triangularis | 0.006 | 0.941 | 0.989 | 1.891 | 0.171 | 0.519 | | 0.151 | 0.698 | 0.816 |
| R middle frontal gyrus | 2.056 | 0.153 | 0.989 | 1.323 | 0.251 | 0.522 | | 2.449 | 0.119 | 0.442 |
| R superior frontal gyrus | 0.615 | 0.434 | 0.989 | 3.194 | 0.075 | 0.302 | | 0.080 | 0.777 | 0.882 |
| R long insular gyrus | 0.116 | 0.734 | 0.989 | 1.386 | 0.241 | 0.522 | | 1.223 | 0.270 | 0.501 |
| R short insular gyrus | 0.045 | 0.831 | 0.989 | 0.190 | 0.663 | 0.741 | | 2.402 | 0.123 | 0.442 |
| R middle occipital gyrus | 0.255 | 0.614 | 0.989 | 2.742 | 0.099 | 0.351 | | 6.234 | 0.013 | 0.114 |
| R superior occipital gyrus | 0.184 | 0.668 | 0.989 | 0.636 | 0.426 | 0.615 | | 1.251 | 0.265 | 0.501 |
| R lateral occipito-temporal gyrus | 0.138 | 0.710 | 0.989 | 0.064 | 0.801 | 0.870 | | 0.459 | 0.499 | 0.689 |
| R lingual gyrus | 0.229 | 0.633 | 0.989 | 3.618 | 0.059 | 0.281 | | 0.205 | 0.651 | 0.811 |
| R parahippocampal gyrus | 1.869 | 0.173 | 0.989 | 0.293 | 0.589 | 0.678 | | 0.026 | 0.873 | 0.961 |
| R orbital gyrus | 0.001 | 0.976 | 0.989 | 3.530 | 0.062 | 0.281 | | 5.762 | 0.017 | 0.120 |
| R angular gyrus | 1.466 | 0.227 | 0.989 | 0.005 | 0.944 | 0.957 | | 0.441 | 0.508 | 0.689 |
| R supramarginal gyrus | 3.496 | 0.063 | 0.782 | 1.384 | 0.241 | 0.522 | | 0.988 | 0.321 | 0.517 |

| | | | | | | | | | | | | |
|---|---|---|---|---|---|---|---|---|---|---|---|---|
| R superior parietal lobule | 1.125 | 0.290 | 0.989 | | 3.661 | 0.057 | 0.281 | | 4.09 | 0.045 | 0.226 | |
| R postcentral gyrus | 1.742 | 0.189 | 0.989 | | 0.345 | 0.558 | 0.669 | | 2.338 | 0.128 | 0.442 | |
| R precentral gyrus | 0.035 | 0.852 | 0.989 | | 3.577 | 0.060 | 0.281 | | 0.875 | 0.351 | 0.523 | |
| R precuneus | 0.283 | 0.595 | 0.989 | | 1.559 | 0.213 | 0.522 | | 5.942 | 0.016 | 0.119 | |
| R straight gyrus | 9.341 | 0.003 | 0.194 | MDD < HC | 0.008 | 0.928 | 0.953 | | 7.235 | 0.008 | 0.099 | |
| R subcallosal gyrus | 0.431 | 0.512 | 0.989 | | 0.505 | 0.478 | 0.627 | | <0.001 | 0.986 | 0.986 | |
| R anterior transverse temporal gyrus | 1.690 | 0.195 | 0.989 | | 8.331 | 0.004 | 0.110 | CC > CT + TT | 2.441 | 0.120 | 0.442 | |
| R lateral superior temporal gyrus | 0.815 | 0.368 | 0.989 | | 9.620 | 0.002 | 0.084 | CC > CT + TT | 1.213 | 0.272 | 0.501 | |
| R planum polare | 0.062 | 0.804 | 0.989 | | 1.270 | 0.261 | 0.522 | | 1.448 | 0.230 | 0.501 | |
| R planum temporale | 3.540 | 0.061 | 0.782 | | 1.689 | 0.195 | 0.522 | | 3.830 | 0.052 | 0.246 | |
| R inferior temporal gyrus | 0.909 | 0.342 | 0.989 | | 3.503 | 0.063 | 0.281 | | 2.228 | 0.137 | 0.453 | |
| R middle temporal gyrus | <0.001 | 0.983 | 0.989 | | 0.979 | 0.324 | 0.559 | | 0.165 | 0.685 | 0.816 | |
| ***cortical thickness*** | | | | | | | | | | | | |
| L frontomarginal gyrus | 4.017 | 0.046 | 0.359 | MDD < HC | 3.745 | 0.054 | 0.425 | | 0.002 | 0.963 | 0.984 | |
| L inferior occipital gyrus | 0.918 | 0.339 | 0.584 | | 0.084 | 0.772 | 0.939 | | 2.191 | 0.140 | 0.898 | |
| L paracentral lobule | 0.009 | 0.926 | 0.985 | | 0.122 | 0.727 | 0.937 | | 0.160 | 0.689 | 0.935 | |
| L subcentral gyrus | 0.952 | 0.330 | 0.584 | | 3.615 | 0.059 | 0.425 | | 0.214 | 0.644 | 0.906 | |
| L transverse frontopolar gyrus | 3.378 | 0.068 | 0.362 | | 1.780 | 0.184 | 0.607 | | 0.500 | 0.480 | 0.898 | |
| L anterior cingulate gyrus | 3.077 | 0.081 | 0.362 | | 2.404 | 0.123 | 0.518 | | 0.111 | 0.739 | 0.943 | |
| L anterior mid-cingulate gyrus | 0.176 | 0.676 | 0.812 | | 0.095 | 0.759 | 0.939 | | 0.243 | 0.622 | 0.906 | |
| L mid-posterior cingulate gyrus | 0.483 | 0.488 | 0.686 | | 1.495 | 0.223 | 0.652 | | 1.094 | 0.297 | 0.898 | |
| L dorsal posterior cingulate gyrus | 0.336 | 0.563 | 0.725 | | 0.063 | 0.802 | 0.939 | | 9.233 | 0.003 | 0.206 | |
| L ventral posterior cingulate gyrus | 0.666 | 0.415 | 0.631 | | 0.016 | 0.898 | 0.950 | | 0.014 | 0.906 | 0.956 | |
| L cuneus | 0.308 | 0.580 | 0.734 | | 0.001 | 0.979 | 0.979 | | 0.220 | 0.640 | 0.906 | |

| | | | | | | | | | | | |
|---|---|---|---|---|---|---|---|---|---|---|---|
| L pars opercularis | 2.759 | 0.098 | 0.374 | | 0.826 | 0.365 | 0.749 | | 0.027 | 0.869 | 0.943 |
| L pars orbitalis | 1.205 | 0.274 | 0.565 | | 0.369 | 0.544 | 0.817 | | 3.163 | 0.077 | 0.898 |
| L pars triangularis | 1.162 | 0.282 | 0.565 | | 2.635 | 0.106 | 0.504 | | 1.130 | 0.289 | 0.898 |
| L middle frontal gyrus | 2.174 | 0.142 | 0.469 | | 0.914 | 0.340 | 0.718 | | 0.083 | 0.774 | 0.943 |
| L superior frontal gyrus | 1.637 | 0.202 | 0.518 | | 0.472 | 0.493 | 0.797 | | 0.052 | 0.820 | 0.943 |
| L long insular gyrus | 0.109 | 0.742 | 0.826 | | 2.003 | 0.159 | 0.574 | | 0.777 | 0.379 | 0.898 |
| L short insular gyrus | 0.154 | 0.695 | 0.812 | | 5.349 | 0.022 | 0.359 | CC > CT + TT | 0.396 | 0.530 | 0.898 |
| L middle occipital gyrus | 1.598 | 0.208 | 0.518 | | 2.671 | 0.104 | 0.504 | | 0.737 | 0.392 | 0.898 |
| L superior occipital gyrus | 2.909 | 0.090 | 0.362 | | 0.26 | 0.611 | 0.871 | | 1.934 | 0.166 | 0.898 |
| L lateral occipito-temporal gyrus | 0.180 | 0.672 | 0.812 | | 1.647 | 0.201 | 0.611 | | 0.767 | 0.382 | 0.898 |
| L lingual gyrus | 0.375 | 0.541 | 0.725 | | 0.057 | 0.812 | 0.939 | | 1.782 | 0.183 | 0.898 |
| L parahippocampal gyrus | 0.572 | 0.450 | 0.655 | | 0.05 | 0.823 | 0.939 | | 1.222 | 0.270 | 0.898 |
| L orbital gyrus | 4.127 | 0.044 | 0.359 | MDD < HC | 0.957 | 0.329 | 0.718 | | 0.077 | 0.782 | 0.943 |
| L angular gyrus | 1.579 | 0.210 | 0.518 | | 0.925 | 0.337 | 0.718 | | 0.475 | 0.492 | 0.898 |
| L supramarginal gyrus | 1.43 | 0.233 | 0.537 | | 1.17 | 0.281 | 0.673 | | 1.323 | 0.251 | 0.898 |
| L superior parietal lobule | 0.126 | 0.723 | 0.826 | | 0.933 | 0.335 | 0.718 | | 3.625 | 0.058 | 0.898 |
| L postcentral gyrus | <0.001 | 0.990 | 1.000 | | 0.752 | 0.387 | 0.751 | | 0.032 | 0.859 | 0.943 |
| L precentral gyrus | 0.159 | 0.691 | 0.812 | | 0.013 | 0.909 | 0.950 | | 1.362 | 0.245 | 0.898 |
| L precuneus | 0.727 | 0.395 | 0.613 | | 0.009 | 0.925 | 0.950 | | 1.392 | 0.240 | 0.898 |
| L straight gyrus | 2.287 | 0.132 | 0.456 | | 0.074 | 0.786 | 0.939 | | <0.001 | 0.984 | 0.984 |
| L subcallosal gyrus | 0.052 | 0.820 | 0.890 | | 1.177 | 0.279 | 0.673 | | 1.535 | 0.217 | 0.898 |
| L anterior transverse temporal gyrus | <0.001 | 1.000 | 1.000 | | 4.347 | 0.038 | 0.425 | CC > CT + TT | 0.372 | 0.543 | 0.898 |
| L lateral superior temporal gyrus | 0.893 | 0.346 | 0.584 | | 3.539 | 0.061 | 0.425 | | 0.066 | 0.797 | 0.943 |
| L planum polare | 4.531 | 0.035 | 0.359 | MDD < HC | 2.071 | 0.152 | 0.574 | | 0.863 | 0.354 | 0.898 |

| | | | | | | | | | | | | |
|---|---|---|---|---|---|---|---|---|---|---|---|---|
| L planum temporale | 1.108 | 0.294 | 0.573 | | 0.184 | 0.668 | 0.907 | | 0.521 | 0.471 | 0.898 |
| L inferior temporal gyrus | 0.556 | 0.457 | 0.655 | | 2.97 | 0.086 | 0.504 | | 0.473 | 0.492 | 0.898 |
| L middle temporal gyrus | 2.975 | 0.086 | 0.362 | | 2.449 | 0.119 | 0.518 | | 0.058 | 0.809 | 0.943 |
| R frontomarginal gyrus | 5.026 | 0.026 | 0.331 | MDD < HC | 1.718 | 0.192 | 0.607 | | 0.351 | 0.554 | 0.898 |
| R inferior occipital gyrus | 1.969 | 0.162 | 0.514 | | 0.106 | 0.745 | 0.939 | | 0.577 | 0.448 | 0.898 |
| R paracentral lobule | 0.102 | 0.750 | 0.826 | | 0.024 | 0.876 | 0.950 | | 0.399 | 0.528 | 0.898 |
| R subcentral gyrus | 0.770 | 0.381 | 0.604 | | 9.272 | 0.003 | 0.202 | CC > CT + TT | 0.452 | 0.502 | 0.898 |
| R transverse frontopolar gyrus | 6.341 | 0.013 | 0.331 | MDD < HC | 1.416 | 0.236 | 0.653 | | 0.231 | 0.631 | 0.906 |
| R anterior cingulate gyrus | 11.909 | 0.001 | 0.052 | MDD < HC | 0.639 | 0.425 | 0.751 | | 0.033 | 0.856 | 0.943 |
| R anterior mid-cingulate gyrus | 3.232 | 0.074 | 0.362 | | 0.44 | 0.508 | 0.804 | | 0.637 | 0.426 | 0.898 |
| R mid-posterior cingulate gyrus | 0.855 | 0.356 | 0.588 | | 0.15 | 0.699 | 0.915 | | 1.547 | 0.215 | 0.898 |
| R dorsal posterior cingulate gyrus | 0.002 | 0.960 | 0.997 | | 0.002 | 0.966 | 0.979 | | 4.719 | 0.031 | 0.898 |
| R ventral posterior cingulate gyrus | 5.286 | 0.023 | 0.331 | MDD < HC | 0.039 | 0.843 | 0.939 | | 1.549 | 0.215 | 0.898 |
| R cuneus | 0.769 | 0.382 | 0.604 | | 0.362 | 0.548 | 0.817 | | <0.001 | 0.984 | 0.984 |
| R pars opercularis | 5.459 | 0.020 | 0.331 | MDD < HC | 6.575 | 0.011 | 0.281 | CC > CT + TT | 0.473 | 0.492 | 0.898 |
| R pars orbitalis | 0.001 | 0.971 | 0.997 | | 2.681 | 0.103 | 0.504 | | 0.696 | 0.405 | 0.898 |
| R pars triangularis | 3.525 | 0.062 | 0.362 | | 1.793 | 0.182 | 0.607 | | 0.257 | 0.613 | 0.906 |
| R middle frontal gyrus | 2.585 | 0.110 | 0.396 | | 0.715 | 0.399 | 0.751 | | 0.434 | 0.511 | 0.898 |
| R superior frontal gyrus | 3.757 | 0.054 | 0.362 | | 0.649 | 0.422 | 0.751 | | 2.241 | 0.136 | 0.898 |
| R long insular gyrus | 2.895 | 0.090 | 0.362 | | 0.734 | 0.393 | 0.751 | | 0.543 | 0.462 | 0.898 |
| R short insular gyrus | 1.370 | 0.243 | 0.544 | | 3.635 | 0.058 | 0.425 | | 0.24 | 0.625 | 0.906 |
| R middle occipital gyrus | 0.955 | 0.330 | 0.584 | | 0.562 | 0.454 | 0.763 | | 0.758 | 0.385 | 0.898 |
| R superior occipital gyrus | 0.359 | 0.550 | 0.725 | | 1.336 | 0.249 | 0.653 | | 0.051 | 0.822 | 0.943 |
| R lateral occipito-temporal gyrus | 0.998 | 0.319 | 0.584 | | 0.544 | 0.462 | 0.763 | | 0.100 | 0.752 | 0.943 |

| | | | | | | | | | | | | |
|---|---|---|---|---|---|---|---|---|---|---|---|---|
| R lingual gyrus | 0.36 | 0.549 | 0.725 | | 0.595 | 0.441 | 0.762 | | 0.349 | 0.555 | 0.898 |
| R parahippocampal gyrus | 1.184 | 0.278 | 0.565 | | 3.734 | 0.055 | 0.425 | | 1.127 | 0.290 | 0.898 |
| R orbital gyrus | 1.530 | 0.218 | 0.518 | | 0.373 | 0.542 | 0.817 | | 0.947 | 0.332 | 0.898 |
| R angular gyrus | 0.994 | 0.320 | 0.584 | | 0.035 | 0.852 | 0.939 | | 0.117 | 0.733 | 0.943 |
| R supramarginal gyrus | 3.989 | 0.047 | 0.359 | MDD < HC | 2.297 | 0.131 | 0.525 | | 0.225 | 0.636 | 0.906 |
| R superior parietal lobule | 0.351 | 0.555 | 0.725 | | 0.010 | 0.922 | 0.950 | | 0.449 | 0.504 | 0.898 |
| R postcentral gyrus | 0.572 | 0.450 | 0.655 | | 0.667 | 0.415 | 0.751 | | 0.014 | 0.905 | 0.956 |
| R precentral gyrus | 1.258 | 0.264 | 0.565 | | 0.243 | 0.623 | 0.871 | | 3.210 | 0.075 | 0.898 |
| R precuneus | 0.241 | 0.624 | 0.777 | | 1.366 | 0.244 | 0.653 | | 0.606 | 0.437 | 0.898 |
| R straight gyrus | 5.931 | 0.016 | 0.331 | MDD < HC | 0.232 | 0.630 | 0.871 | | 0.408 | 0.524 | 0.898 |
| R subcallosal gyrus | 1.887 | 0.171 | 0.518 | | 0.169 | 0.681 | 0.908 | | 0.773 | 0.380 | 0.898 |
| R anterior transverse temporal gyrus | 0.007 | 0.933 | 0.985 | | 7.122 | 0.008 | 0.281 | CC > CT + TT | 0.134 | 0.714 | 0.943 |
| R lateral superior temporal gyrus | 1.579 | 0.210 | 0.518 | | 3.115 | 0.079 | 0.501 | | 0.001 | 0.978 | 0.984 |
| R planum polare | 0.110 | 0.740 | 0.826 | | 0.322 | 0.571 | 0.834 | | 0.641 | 0.424 | 0.898 |
| R planum temporale | 3.537 | 0.062 | 0.362 | | 5.203 | 0.024 | 0.359 | CC > CT + TT | 0.034 | 0.854 | 0.943 |
| R inferior temporal gyrus | 1.528 | 0.218 | 0.518 | | 1.158 | 0.283 | 0.673 | | 1.054 | 0.306 | 0.898 |
| R middle temporal gyrus | 1.545 | 0.215 | 0.518 | | 0.038 | 0.845 | 0.939 | | 0.178 | 0.674 | 0.931 |
| ***Subcortical volume*** | | | | | | | | | | | |
| L thalamus | 1.614 | 0.205 | 0.719 | | 2.215 | 0.138 | 0.387 | | 0.438 | 0.509 | 0.794 |
| L caudate nucleus | 2.068 | 0.152 | 0.710 | | 5.446 | 0.021 | 0.251 | CC > CT + TT | 0.408 | 0.524 | 0.794 |
| L putamen | 0.389 | 0.534 | 0.934 | | 0.107 | 0.744 | 0.801 | | 4.757 | 0.030 | 0.213 |
| L globus pallidum | 0.001 | 0.973 | 0.973 | | 1.686 | 0.196 | 0.457 | | 0.241 | 0.624 | 0.794 |
| L hippocampus | 0.100 | 0.753 | 0.973 | | 0.903 | 0.343 | 0.499 | | 0.269 | 0.604 | 0.794 |
| L amygdala | 0.861 | 0.355 | 0.828 | | 2.219 | 0.138 | 0.387 | | 0.019 | 0.890 | 0.890 |

| | | | | | | | | | | | |
|---|---|---|---|---|---|---|---|---|---|---|---|
| L nucleus accumbens | 0.166 | 0.684 | 0.973 | | 0.275 | 0.601 | 0.764 | | 0.830 | 0.363 | 0.794 |
| R thalamus | 4.193 | 0.042 | 0.587 | MDD < HC | 1.046 | 0.308 | 0.499 | | 1.591 | 0.209 | 0.671 |
| R caudate nucleus | 2.210 | 0.139 | 0.710 | | 4.468 | 0.036 | 0.251 | CC > CT + TT | 1.392 | 0.240 | 0.671 |
| R putamen | 0.008 | 0.928 | 0.973 | | 0.870 | 0.352 | 0.499 | | 5.806 | 0.017 | 0.213 |
| R globus pallidum | 0.601 | 0.439 | 0.878 | | 3.189 | 0.076 | 0.353 | | 0.411 | 0.522 | 0.794 |
| R hippocampus | 0.989 | 0.321 | 0.828 | | 0.049 | 0.826 | 0.826 | | 0.081 | 0.776 | 0.836 |
| R amygdala | 0.033 | 0.857 | 0.973 | | 0.856 | 0.356 | 0.499 | | 0.105 | 0.746 | 0.836 |
| R nucleus accumbens | 0.009 | 0.926 | 0.973 | | 0.190 | 0.663 | 0.774 | | 3.627 | 0.058 | 0.272 |

The F and P values were obtained using analysis of covariance (ANCOVA) adjusted for age, gender, education level, medication, and total intracranial volume as covariates

The false discovery rate (FDR) was applied in each analysis for multiple comparison correction, q < 0.05; cortical volume: 76 comparisons in both hemispheres, cortical thickness: 76 comparisons in both hemispheres; subcortical volume: 14 comparisons in both hemispheres.

*Regions that remained significant after the multiple comparison correction are marked with an asterisk.

MDD, major depressive disorder; HC, healthy controls; CC, CC genotype of rs1360780; CT + TT, CT or TT genotype of rs1360780; L, left hemisphere; R, right hemisphere. $P_{uncorr}$, uncorrected p-value; $P_{corr}$, FDR-corrected p-value.

Table S4. Summary of post-hoc analyses of gray-matter volumes or cortical thickness with significant diagnosis-by-genotype interactions in patients with major depressive disorder and healthy controls.

| brain regions | **MDD (n = 114)** | | | | **HC (n = 88)** | | | |
|---|---|---|---|---|---|---|---|---|
| | CC (n = 72) | CT + TT (n = 42) | F | $P_{uncorr}$ | CC (n = 58) | CT + TT (n = 30) | F | $P_{uncorr}$ |
| ***cortical volume*** | | | | | | | | |
| L pars triangularis | 2564.49 ± 613.39 | 2303.79 ± 559.81 | 9.353 | 0.003 | 2575.86 ± 571.00 | 2750.63 ± 597.15 | 3.437 | 0.067 |
| L supramarginal gyrus | 6690.22 ± 1204.64 | 6168.60 ± 1276.03 | 11.172 | 0.001 | 6569.19 ± 1126.12 | 6809.53 ± 820.78 | 1.885 | 0.173 |
| L superior parietal lobule | 6393.49 ± 1089.78 | 5872.24 ± 804.03 | 14.819 | <0.001 | 6207.41 ± 932.16 | 6423.77 ± 876.62 | 2.144 | 0.147 |
| L posterior mid-cingulate gyrus | 2628.75 ± 448.17 | 2557.38 ± 377.95 | 3.732 | 0.056 | 2608.38 ± 427.79 | 2775.50 ± 484.42 | 5.113 | 0.026 |
| R frontomarginal gyrus | 1721.94 ± 330.36 | 1583.38 ± 315.56 | 9.815 | 0.002 | 1715.86 ± 337.46 | 1800.43 ± 342.20 | 1.591 | 0.211 |

Data are mean ± standard deviation ($mm^3$).

Only cortical regions with significant diagnosis-by-genotype interactions after the multiple comparison correction are included in the table (FDR-corrected $P < 0.05$).

The F and P values were obtained using analysis of covariance (ANCOVA) adjusted for age, gender, education level, medication, and total intracranial volume as covariates

MDD, major depressive disorder; HC, healthy controls; CC, CC genotype of rs1360780; CT + TT, CT or TT genotype of rs1360780; L, left hemisphere; R, right hemisphere; $P_{uncorr,}$ uncorrected p-value.

Table S5. Post-hoc analysis of cortical volumes with significant diagnosis-by-genotype interactions in patients with major depressive disorder and healthy controls.

| Cortical regions | MDD (n = 114) | | | | HC (n = 88) | | | |
|---|---|---|---|---|---|---|---|---|
| | CC (n = 72) | CT + TT (n = 42) | F | $P_{uncorr}$ | CC (n = 58) | CT + TT (n = 30) | F | $P_{uncorr}$ |
| L pars triangularis | 2564.49 ± 613.39 | 2303.79 ± 559.81 | 9.353 | 0.003 | 2575.86 ± 571.00 | 2750.63 ± 597.15 | 3.437 | 0.067 |
| L short insular gyrus | 2061.24 ± 339.47 | 2009.71 ± 306.35 | 4.299 | 0.041 | 2153.90 ± 359.80 | 2222.77 ± 273.86 | 0.918 | 0.341 |
| L middle occipital gyrus | 4827.39 ± 867.23 | 4655.98 ± 715.51 | 4.049 | 0.047 | 4960.03 ± 904.58 | 5176.83 ± 963.02 | 1.605 | 0.209 |
| L supramarginal gyrus | 6690.22 ± 1204.64 | 6168.60 ± 1276.03 | 11.172 | 0.001 | 6569.19 ± 1126.12 | 6809.53 ± 820.78 | 1.885 | 0.173 |
| L superior parietal lobule | 6393.49 ± 1089.78 | 5872.24 ± 804.03 | 14.819 | <0.001 | 6207.41 ± 932.16 | 6423.77 ± 876.62 | 2.144 | 0.147 |
| L precuneus | 5857.99 ± 973.70 | 5624.14 ± 731.20 | 6.956 | 0.010 | 5743.05 ± 1010.20 | 5905.17 ± 675.48 | 0.927 | 0.339 |
| L posterior mid-cingulate gyrus | 2628.75 ± 448.17 | 2557.38 ± 377.95 | 3.732 | 0.056 | 2608.38 ± 427.79 | 2775.50 ± 484.42 | 5.113 | 0.026 |
| R frontomarginal gyrus | 1721.94 ± 330.36 | 1583.38 ± 315.56 | 9.815 | 0.002 | 1715.86 ± 337.46 | 1800.43 ± 342.20 | 1.591 | 0.211 |
| R transverse frontopolar gyrus | 2310.22 ± 406.80 | 2163.14 ± 405.36 | 4.688 | 0.033 | 2270.14 ± 357.84 | 2361.30 ± 454.03 | 1.252 | 0.266 |
| R dorsal posterior cingulate gyrus | 1432.22 ± 336.10 | 1375.24 ± 323.90 | 3.974 | 0.049 | 1403.14 ± 303.86 | 1478.63 ± 332.67 | 1.914 | 0.170 |
| R middle occipital gyrus | 5291.47 ± 1090.42 | 4997.19 ± 941.62 | 8.417 | 0.005 | 5298.98 ± 931.80 | 5379.43 ± 933.44 | 0.535 | 0.466 |
| R orbital gyrus | 5980.08 ± 877.20 | 5722.10 ± 889.00 | 8.279 | 0.005 | 6046.02 ± 812.76 | 6022.37 ± 723.25 | 0.049 | 0.826 |
| R superior parietal lobule | 4902.31 ± 762.04 | 4623.38 ± 756.98 | 6.965 | 0.010 | 4969.45 ± 818.88 | 4928.53 ± 609.30 | 0.001 | 0.972 |
| R precuneus | 5748.88 ± 951.95 | 5502.71 ± 817.23 | 7.430 | 0.007 | 5757.43 ± 898.57 | 5879.60 ± 937.58 | 0.606 | 0.438 |
| R straight gyrus | 1655.61 ± 267.93 | 1606.14 ± 266.42 | 4.070 | 0.046 | 1713.90 ± 259.79 | 1800.77 ± 295.04 | 3.335 | 0.071 |

Data are mean ± standard deviation ($mm^3$).

Only cortical regions with significant diagnosis-by-genotype interactions (uncorrected p-value < 0.05) in the main-analysis are included in the table.

The F and P values were obtained using analysis of covariance (ANCOVA) adjusted for age, gender, education level, medication, and total intracranial volume as

covariates

MDD, major depressive disorder; HC, healthy controls; CC, CC genotype of rs1360780; CT + TT, CT or TT genotype of rs1360780; L, left hemisphere; R, right hemisphere; $P_{uncorr,}$ uncorrected P value.

Table S6. Post-hoc analysis of subcortical volumes with significant diagnosis-by-genotype interactions in patients with major depressive disorder and healthy controls.

| Subcortical regions | **MDD (n = 114)** | | | | **HC (n = 88)** | | | |
|---|---|---|---|---|---|---|---|---|
| | CC (n = 72) | CT + TT (n = 42) | F | $P_{uncorr}$ | CC (n = 58) | CT + TT (n = 30) | F | $P_{uncorr}$ |
| L putamen | 5141.98 ± 710.80 | 5106.15 ± 543.83 | 2.725 | 0.102 | 5291.62 ± 763.71 | 5447.75 ± 495.58 | 1.821 | 0.181 |
| R putamen | 5042.27 ± 681.64 | 4940.60 ± 560.81 | 5.049 | 0.027 | 5117.38 ± 739.21 | 5233.06 ± 442.46 | 1.144 | 0.288 |

Data are mean ± standard deviation ($mm^3$).

Only subcortical regions with significant diagnosis-by-genotype interactions (uncorrected p-value < 0.05) in the main-analysis are included in the table.

The F and P values were obtained using analysis of covariance (ANCOVA) adjusted for age, gender, education level, medication, and total intracranial volume as covariates

MDD, major depressive disorder; HC, healthy controls; CC, CC genotype of rs1360780; CT + TT, CT or TT genotype of rs1360780; L, left hemisphere; R, right hemisphere; $P_{uncorr,}$ uncorrected P value.

Table S7. Post-hoc analysis of cortical thickness with significant diagnosis-by-genotype interactions in patients with major depressive disorder and healthy controls.

| Cortical regions | MDD (n = 114) | | | | HC (n = 88) | | | |
|---|---|---|---|---|---|---|---|---|
| | CC (n = 72) | CT + TT (n = 42) | F | $P_{uncorr}$ | CC (n = 58) | CT + TT (n = 30) | F | $P_{uncorr}$ |
| L dorsal posterior cingulate gyrus | 2.746 ± 0.176 | 2.677 ± 0.210 | 4.010 | 0.048 | 2.738 ± 0.169 | 2.815 ± 0.200 | 6.693 | 0.011 |
| R dorsal posterior cingulate gyrus | 2.800 ± 0.170 | 2.757 ± 0.175 | 2.581 | 0.111 | 2.795 ± 0.171 | 2.827 ± 0.167 | 3.128 | 0.081 |

Data are mean ± standard deviation ($mm^3$).

Only brain regions with significant diagnosis-by-genotype interactions (uncorrected p-value < 0.05) in the main-analysis are included in the post-hoc analysis.

The F and P values were obtained using analysis of covariance (ANCOVA) adjusted for age, gender, education level, medication, and total intracranial volume as covariates

MDD, major depressive disorder; HC, healthy controls; CC, CC genotype of rs1360780; CT + TT, CT or TT genotype of rs1360780; L, left hemisphere; R, right hemisphere; $P_{uncorr,}$ uncorrected P value.

Table S8. Difference in DNA methylation of the *FKBP5* gene among groups determined by genotype and diagnosis.

| CpG | MDD (%, n = 104) | HC (%, n = 82) | **MDD vs. HC** | | CC (%, n = 118) | CT + TT (%, n = 68) | **CC vs. CT + TT** | | **Diagnosis x Genotype interaction** | |
|---|---|---|---|---|---|---|---|---|---|---|
| | | | F | p | | | F | p | F | p |
| pos 1 | 97.65 ± 2.57 | 97.10 ± 2.61 | 2.256 | 0.135 | 97.65 ± 2.18 | 96.98 ± 3.17 | 2.168 | 0.143 | 2.570 | 0.111 |
| pos 2 | 90.62 ± 5.37 | 90.49 ± 3.05 | 0.324 | 0.570 | 90.88 ± 4.64 | 90.02 ± 4.20 | 1.228 | 0.269 | 1.094 | 0.297 |

Data are mean ± standard deviation (%).

The F and P values were obtained using analysis of covariance (ANCOVA) adjusted for age, gender, and medication.

MDD, major depressive disorder; HC, healthy controls; CC, participants with CC genotype of rs1360780; CT + TT, participants with CT or TT genotype of rs1360780.

Table S9. Exploratory analysis of DNA methylation of the *FKBP5* gene in groups determined by genotype and diagnosis.

| CpG | **MDD (n = 104)** | | | | **HC (n = 82)** | | | |
|---|---|---|---|---|---|---|---|---|
| | CC (n = 65) | CT + TT (n = 39) | F | p | CC (n = 53) | CT + TT (n = 29) | F | p |
| pos 1 | 98.07 ± 1.77 | 96.94 ± 3.44 | 6.236 | 0.014 | 97.13 ± 2.52 | 97.04 ± 2.82 | <0.001 | 0.994 |
| pos 2 | 91.13 ± 5.51 | 89.77 ± 5.08 | 2.133 | 0.147 | 90.57 ± 3.28 | 90.35 ± 2.64 | 0.164 | 0.686 |

Data are mean ± standard deviation (%).

The F and P values were obtained using analysis of covariance (ANCOVA) adjusted for age, gender, and medication.

MDD, major depressive disorder; HC, healthy controls; CC, participants with CC genotype of rs1360780; CT + TT, participants with CT or TT genotype of rs1360780.

Table S10. Correlation analysis between cortical gray-matter volume and DNA methylation in intron 7 of the *FKBP5* gene among groups determined by diagnosis, genotype, and diagnosis-by-genotype interaction.

| | **Subgroups** | **Regions** | **r or t** | **$P_{uncorr}$** | **CpG** |
|---|---|---|---|---|---|
| Diagnostic groups | MDD (n=104) | R subcallosal gyrus | -0.235 | 0.020 | pos 1 |
| | HC (n=82) | L dorsal posterior cingulate gyrus | -0.412 | <0.001 | pos 1 |
| | | R paracentral lobule | 0.228 | 0.046 | pos 2 |
| | | R pars orbitalis | 0.290 | 0.011 | pos 2 |
| | | R middle frontal gyrus | -0.233 | 0.041 | pos 1 |
| | | R parahippocampal gyrus | -0.338 | 0.003 | pos 2 |
| | | R precuneus | -0.247 | 0.030 | pos 2 |
| | | R straight gyrus | 0.286 | 0.012 | pos 2 |
| Genotype groups | T (n= 68) | L middle frontal gyrus | -0.276 | 0.030 | pos 2 |
| | | L superior frontal gyrus | -0.258 | 0.043 | pos 1 |
| | | L suparmarginal gyrus | -0.291 | 0.022 | pos 1 |
| | | L suparmarginal gyrus | -0.401 | 0.001 | pos 2 |
| | | L precental gyrus | -0.256 | 0.045 | pos 1 |
| | | L precuneus | -0.322 | 0.011 | pos 1 |
| | | L precuneus | -0.382 | 0.002 | pos 2 |
| | | L straight gyrus | -0.252 | 0.048 | pos 1 |
| | | L middle temporal gyrus | -0.263 | 0.039 | pos 1 |
| | | L middle temporal gyrus | -0.267 | 0.036 | pos 2 |
| | | R subcentral gyrus | -0.307 | 0.015 | pos 1 |
| | | R dorsal posterior cingulate gyrus | -0.283 | 0.026 | pos 1 |
| | | R dorsal posterior cingulate gyrus | -0.290 | 0.022 | pos 2 |
| | | R pars orbitalis | -0.288 | 0.023 | pos 1 |
| | | R parahippocampal gyrus | -0.312 | 0.013 | pos 2 |
| | | R precuneus | -0.287 | 0.024 | pos 2 |
| | | R subcallosal gyrus | -0.360 | 0.004 | pos 2 |
| | | R inferior temporal gyrus | -0.258 | 0.043 | pos 1 |
| | CC (n= 118) | L angular gyrus | 0.202 | 0.033 | pos 1 |
| | | L precental gyrus | 0.193 | 0.042 | pos 1 |
| | | R frontomarginal gyrus | 0.186 | 0.049 | pos 1 |
| | | R straight gyrus | 0.218 | 0.021 | pos 1 |
| | | R subcallosal gyrus | -0.216 | 0.022 | pos 1 |
| | | R middle temporal gyrus | 0.190 | 0.044 | pos 1 |

| | | | | | |
|---|---|---|---|---|---|
| Interaction effect | Diagnosis × methylation | L dorsal posterior cingulate gyrus | 2.303 | 0.022 | pos 1 |
| | | L parahippocampal gyrus | 2.157 | 0.032 | pos 2 |
| | | R paracentral lobule | -2.029 | 0.044 | pos 2 |
| | | R pars orbitalis | -2.201 | 0.029 | pos 2 |
| | | R middle frontal gyrus | 2.556 | 0.011 | pos 1 |
| | | R parahippocampal gyrus | 2.461 | 0.015 | pos 2 |
| | | R precuneus | 2.000 | 0.047 | pos 2 |
| | Genotype × methylation | L angular gyrus | -2.137 | 0.034 | pos 1 |
| | | L supramarginal gyrus | -2.311 | 0.022 | pos 2 |
| | | L precentral gyrus | -3.079 | 0.002 | pos 1 |
| | | L straight gyrus | -2.169 | 0.031 | pos 1 |
| | | R subcentral gyrus | -2.900 | 0.004 | pos 1 |
| | | R pars orbitalis | -2.777 | 0.006 | pos 1 |
| | | R straight gyrus | -2.494 | 0.014 | pos 1 |
| | | R subcallosal gyrus | -2.165 | 0.032 | pos 2 |
| | | R middle temporal gyrus | -2.231 | 0.027 | pos 1 |
| | Diagnosis × genotype × methylation | L pars opercularis | -2.114 | 0.036 | pos 2 |
| | | L precentral gyrus | -2.286 | 0.023 | pos 2 |
| | | L planum polare | -2.061 | 0.041 | pos 2 |
| | | R inferior occipital gyrus | -2.558 | 0.011 | pos 1 |
| | | R superior frontal gyrus | -2.087 | 0.038 | pos 1 |
| | | R angular gyrus | -2.397 | 0.018 | pos 1 |
| | | R precuneus | -2.100 | 0.037 | pos 1 |
| | | R precuneus | -2.475 | 0.014 | pos 2 |

A two-tailed Pearson's partial correlation was performed to analyze the correlations between the cortical volumes and percentage of *FKBP5* gene DNA methylation adjusting for following covariates according to each analysis: MDD group: age, gender, education level, medication, total intracranial cavity volume, and *FKBP5* genotype; HC group: age, gender, education level, total intracranial cavity volume, and *FKBP5* genotype; T or CC group: age, gender, education level, medication, total intracranial cavity volume, and diagnosis.

A hierarchical moderated regression analysis was performed to investigated the interactive effect of the DNA methylation with the diagnosis, genotype, or both diagnosis and genotype in terms of the correlation with the cortical volumes.

Only regions with statistically significant correlations are shown ($P_{uncorr} < 0.05$). No cortical regions remained significant after the multiple comparison correction ($P_{corr} > 0.05$).

The FDR was applied in each analysis for multiple comparison correction, q < 0.05; for diagnosis effect (MDD or HC group): 304 comparisons (=38 cortical regions x 2 hemispheres x 2 diagnosis groups x 2 CpG sites); genotype effect (T allele carrier or C homozygote group): 304 comparisons (=38 cortical regions x 2 hemispheres x 2 genotype groups x 2 CpG sites); and interactive effects (e.g. diagnosis-by-methylation,

genotype-by-methylation, diagnosis-by-genotype-by-methylation): 456 comparisons (=38 cortical regions x 2 hemispheres x 3 interactions x 2 CpG sites).

The r-values for Pearson's partial correlation analyses and t-value for the hierarchical moderated regression analyses.

MDD, patients with major depressive disorder; HC, healthy controls; MDD-T, MDD patients with T allele of rs1360780; MDD-CC, MDD patients with CC genotype of rs1360780; L, left hemisphere; R, right hemisphere; $P_{uncorr,}$ uncorrected P value.

Table S11. Correlation analysis between subcortical gray-matter volume and DNA methylation in intron 7 of the *FKBP5* gene among groups determined by diagnosis, genotype, and diagnosis-by-genotype interaction.

| | **Subgroups** | **Regions** | **r or t** | **$P_{uncorr}$** | **CpG** |
|---|---|---|---|---|---|
| Diagnostic groups | HC (n=82) | R globus pallidum | -0.240 | 0.035 | pos 2 |
| | | R hippocampus | -0.226 | 0.048 | pos 2 |
| Genotype groups | T (n= 68) | R amygdala | -0.269 | 0.034 | pos 1 |
| | CC (n= 118) | L Caudate nucleus | 0.198 | 0.037 | pos 1 |
| Interaction effect | Diagnosis × genotype × methylation | R thalamus | -2.484 | 0.014 | pos 1 |

A two-tailed Pearson's partial correlation was performed to analyze the correlations between the subcortical volumes and percentage of *FKBP5* gene DNA methylation adjusting for following covariates according to each analysis: MDD group: age, gender, education level, medication, total intracranial cavity volume, and *FKBP5* genotype; HC group: age, gender, education level, total intracranial cavity volume, and *FKBP5* genotype; T or CC group: age, gender, education level, medication, total intracranial cavity volume, and diagnosis.

A hierarchical moderated regression analysis was performed to investigated the interactive effect of the DNA methylation with the diagnosis, genotype, or both diagnosis and genotype in terms of the correlation with the subcortical volumes.

Only regions with statistically significant correlations are shown ($P_{uncorr} < 0.05$). No subcortical regions remained significant after the multiple comparison correction ($P_{corr} > 0.05$).

The FDR was applied in each analysis for multiple comparison correction, q < 0.05; for diagnosis effect: 56 comparisons (=7 subcortical regions x 2 hemispheres x 2 diagnosis groups x 2 CpG sites); genotype effect: 56 comparisons (=7 subcortical regions x 2 hemispheres x 2 genotype groups x 2 CpG sites); and interactive effects: 84 comparisons (=7 subcortical regions x 2 hemispheres x 3 interactions x 2 CpG sites).

The r-values for Pearson's partial correlation analyses and t-value for the hierarchical moderated regression analyses.

MDD, patients with major depressive disorder; HC, healthy controls; MDD-T, MDD patients with T allele of rs1360780; MDD-CC, MDD patients with CC genotype of rs1360780; L, left hemisphere; R, right hemisphere; $P_{uncorr,}$ uncorrected P value.

Table S12. Correlation analysis between cortical thickness and DNA methylation in intron 7 of the *FKBP5* gene among groups determined by diagnosis, genotype, and diagnosis-by-genotype interaction.

| | **Subgroups** | **Regions** | **r** | $\mathbf{P_{uncorr}}$ | **CpG** |
|---|---|---|---|---|---|
| Diagnostic groups | MDD (n=104) | L inferior occipital gyrus | 0.253 | 0.012 | pos 1 |
| | | L transverse frontopolar gyrus | 0.246 | 0.015 | pos 1 |
| | | L dorsal posterior cingulate gyrus | 0.215 | 0.033 | pos 1 |
| | | L middle occipital gyrus | 0.281 | 0.005 | pos 1 |
| | | L superior occipital gyrus | 0.230 | 0.023 | pos 1 |
| | | L lingual gyrus | 0.205 | 0.043 | pos 1 |
| | | L angular gyrus | 0.206 | 0.042 | pos 1 |
| | | L precuneus | 0.210 | 0.038 | pos 1 |
| | | R frontomarginal gyrus | 0.248 | 0.014 | pos 1 |
| | | R middle occipital gyrus | 0.209 | 0.039 | pos 1 |
| | | R angular gyrus | 0.217 | 0.032 | pos 1 |
| | HC (n=82) | L pars opercularis | 0.307 | 0.007 | pos 1 |
| | | L fusiform gyrus | 0.251 | 0.028 | pos 1 |
| | | L suparmarginal gyrus | 0.225 | 0.049 | pos 1 |
| | | L inferior temporal gyrus | 0.303 | 0.007 | pos 1 |
| | | R inferior occipital gyrus | 0.234 | 0.041 | pos 1 |
| | | R pars opercularis | 0.266 | 0.020 | pos 1 |
| | | R fusiform gyrus | 0.271 | 0.017 | pos 1 |
| | | R postcentral gyrus | 0.231 | 0.043 | pos 1 |
| | | R subcallosal gyrus | -0.282 | 0.013 | pos 1 |
| | | R inferior temporal gyrus | 0.241 | 0.035 | pos 1 |
| | | R middle temporal gyrus | 0.256 | 0.025 | pos 1 |
| Genotype groups | T (n= 68) | L subcentral gyrus | -0.259 | 0.042 | pos 1 |
| | | R frontomarginal gyrus | 0.271 | 0.033 | pos 2 |
| | | R ventral posterior cingulate gyrus | 0.352 | 0.005 | pos 1 |
| | | R ventral posterior cingulate gyrus | 0.305 | 0.016 | pos 2 |
| | CC (n= 118) | L pars opercularis | 0.258 | 0.006 | pos 1 |
| | | L middle frontal gyrus | -0.190 | 0.044 | pos 2 |
| | | L superior frontal gyrus | 0.257 | 0.006 | pos 1 |
| | | L middle occipital gyrus | 0.247 | 0.009 | pos 1 |
| | | L superior occipital gyrus | 0.239 | 0.011 | pos 1 |
| | | L fusiform gyrus | 0.269 | 0.004 | pos 1 |
| | | L parahippocampal gyrus | 0.255 | 0.007 | pos 1 |

| | | | | | |
|---|---|---|---|---|---|
| | | L angular gyrus | 0.282 | 0.003 | pos 1 |
| | | L angular gyrus | -0.247 | 0.009 | pos 2 |
| | | L supramarginal gyrus | 0.216 | 0.022 | pos 1 |
| | | L supramarginal gyrus | -0.229 | 0.015 | pos 2 |
| | | L superior parietal lobule | -0.231 | 0.014 | pos 2 |
| | | L precuneus | 0.207 | 0.028 | pos 1 |
| | | L precuneus | -0.220 | 0.020 | pos 2 |
| | | L inferior temporal gyrus | 0.210 | 0.026 | pos 1 |
| | | L middle temporal gyrus | 0.213 | 0.024 | pos 1 |
| | | R inferior occipital gyrus | 0.244 | 0.010 | pos 1 |
| | | R paracentral lobule | 0.191 | 0.044 | pos 1 |
| | | R transverse frontopolar gyrus | 0.353 | **<0.001*** | pos 1 |
| | | R pars opercularis | 0.291 | 0.002 | pos 1 |
| | | R pars orbitalis | 0.235 | 0.013 | pos 1 |
| | | R superior frontal gyrus | 0.288 | 0.002 | pos 1 |
| | | R short insular gyrus | 0.253 | 0.007 | pos 1 |
| | | R middle occipital gyrus | 0.309 | 0.001 | pos 1 |
| | | R fusiform gyrus | 0.298 | 0.001 | pos 1 |
| | | R orbital gyrus | 0.266 | 0.005 | pos 1 |
| | | R angular gyrus | 0.197 | 0.038 | pos 1 |
| | | R supramarginal gyrus | -0.203 | 0.032 | pos 2 |
| | | R precentral gyrus | 0.198 | 0.037 | pos 1 |
| | | R precuneus | -0.271 | 0.004 | pos 2 |
| | | R lateral superior temporal gyrus | 0.210 | 0.027 | pos 1 |
| | | R inferior temporal gyrus | 0.215 | 0.023 | pos 1 |
| | | R middle temporal gyrus | 0.315 | 0.001 | pos 1 |
| Interaction effects | Diagnosis × methylation | L frontomarginal gyrus | 2.675 | 0.008 | pos 1 |
| | | L inferior occipital gyrus | 2.181 | 0.031 | pos 1 |
| | | L dorsal posterior cingulate gyrus | 2.202 | 0.029 | pos 1 |
| | | R subcallosal gyrus | 2.016 | 0.045 | pos 1 |
| | Genotype × methylation | L transverse frontopolar gyrus | 2.632 | 0.009 | pos 2 |
| | | L pars triangularis | 2.435 | 0.016 | pos 2 |
| | | L superior occipital gyrus | 2.245 | 0.026 | pos 2 |
| | | L lingual gyrus | 2.480 | 0.014 | pos 2 |
| | | L angular gyrus | -2.061 | 0.041 | pos 1 |
| | | L angular gyrus | 2.351 | 0.020 | pos 2 |
| | | L supramarginal gyrus | 2.152 | 0.033 | pos 2 |

| | | | | | |
|---|---|---|---|---|---|
| | | L postcentral gyrus | 2.605 | 0.010 | pos 2 |
| | | L precentral gyrus | -2.148 | 0.033 | pos 1 |
| | | L middle temporal gyrus | 2.132 | 0.034 | pos 2 |
| | | R frontomarginal gyrus | 2.804 | 0.006 | pos 2 |
| | | R transverse frontopolar gyrus | -2.486 | 0.014 | pos 1 |
| | | R ventral posterior cingulate gyrus | 2.308 | 0.022 | pos 1 |
| | | R ventral posterior cingulate gyrus | 3.142 | 0.002 | pos 2 |
| | | R pars opercularis | -2.099 | 0.037 | pos 1 |
| | | R pars orbitalis | -2.785 | 0.006 | pos 1 |
| | | R superior frontal gyrus | -2.063 | 0.041 | pos 1 |
| | | R short insular gyrus | -2.086 | 0.038 | pos 1 |
| | | R middle occipital gyrus | 2.231 | 0.027 | pos 2 |
| | | R fusiform gyrus | -2.080 | 0.039 | pos 1 |
| | | R orbital gyrus | -2.213 | 0.028 | pos 1 |
| | | R precentral gyrus | -2.319 | 0.022 | pos 1 |
| | Diagnosis × genotype × methylation | L supramarginal gyrus | -2.323 | 0.021 | pos 1 |

A two-tailed Pearson's partial correlation was performed to analyze the correlations between the cortical thickness and percentage of *FKBP5* gene DNA methylation adjusting for following covariates according to each analysis: MDD group: age, gender, education level, medication, total intracranial cavity volume, and *FKBP5* genotype; HC group: age, gender, education level, total intracranial cavity volume, and *FKBP5* genotype; T or CC group: age, gender, education level, medication, total intracranial cavity volume, and diagnosis.

A hierarchical moderated regression analysis was performed to investigated the interactive effect of the DNA methylation with the diagnosis, genotype, or both diagnosis and genotype in terms of the correlation with the cortical thickness.

Only regions with statistically significant correlations are shown ($P_{uncorr} < 0.05$).

*Regions that remained significant after the multiple comparison correction are marked with an asterisk.

The FDR was applied in each analysis for multiple comparison correction, q < 0.05; for diagnosis effect (MDD or HC group): 304 comparisons (=38 cortical regions x 2 hemispheres x 2 diagnosis groups x 2 CpG sites); genotype effect (T allele carrier or C homozygote group): 304 comparisons (=38 cortical regions x 2 hemispheres x 2 genotype groups x 2 CpG sites); and interactive effects (e.g. diagnosis-by-methylation, genotype-by-methylation, diagnosis-by-genotype-by-methylation): 456 comparisons (=38 cortical regions x 2 hemispheres x 3 interactions x 2 CpG sites).

The r-values for Pearson's partial correlation analyses and t-value for the hierarchical moderated regression analyses.

MDD, patients with major depressive disorder; HC, healthy controls; MDD-T, MDD patients with T allele of rs1360780; MDD-CC, MDD patients with CC genotype of rs1360780; L, left hemisphere; R, right hemisphere;

$P_{uncorr,}$ uncorrected P value.

Table S13. Exploratory analysis investigating effect of diagnosis, genotype, and diagnosis-by-genotype interaction on cortical surface area of whole-brain.

| brain regions | MDD vs. HC | | | CC vs. CT + TT | | | Diagnosis x Genotype interaction | | |
|---|---|---|---|---|---|---|---|---|---|
| | F | $P_{uncorr}$ | $P_{corr}$ | F | $P_{uncorr}$ | $P_{corr}$ | F | $P_{uncorr}$ | $P_{corr}$ |
| ***Cortical surface area*** | | | | | | | | | |
| L frontomarginal gyrus | 0.223 | 0.638 | 0.905 | 1.860 | 0.174 | 0.977 | 0.858 | 0.355 | 0.808 |
| L inferior occipital gyrus | 0.034 | 0.853 | 0.958 | 0.049 | 0.826 | 0.977 | 4.023 | 0.046 | 0.354 |
| L paracentral lobule | 3.109 | 0.079 | 0.549 | 0.544 | 0.461 | 0.977 | 0.531 | 0.467 | 0.808 |
| L subcentral gyrus | 0.064 | 0.801 | 0.947 | 2.146 | 0.145 | 0.977 | 0.035 | 0.851 | 0.951 |
| L transverse frontopolar gyrus | 1.655 | 0.200 | 0.758 | 0.073 | 0.787 | 0.977 | 1.681 | 0.196 | 0.711 |
| L anterior cingulate gyrus | 0.326 | 0.569 | 0.905 | 0.096 | 0.757 | 0.977 | 0.676 | 0.412 | 0.808 |
| L anterior midcingulate gyrus | 0.750 | 0.388 | 0.866 | 0.064 | 0.800 | 0.977 | 0.225 | 0.636 | 0.838 |
| L posterior midcingulate gyrus | 1.096 | 0.297 | 0.758 | 0.913 | 0.340 | 0.977 | 0.373 | 0.542 | 0.808 |
| L dorsal posterior cingulate gyrus | 3.459 | 0.064 | 0.549 | 1.804 | 0.181 | 0.977 | 4.010 | 0.047 | 0.354 |
| L ventral posterior cingulate gyrus | 1.340 | 0.248 | 0.758 | 0.216 | 0.643 | 0.977 | 0.469 | 0.494 | 0.808 |
| L cuneus | 1.252 | 0.265 | 0.758 | 0.152 | 0.697 | 0.977 | 0.607 | 0.437 | 0.808 |
| L pars opercularis | 0.976 | 0.324 | 0.771 | 0.045 | 0.833 | 0.977 | 0.449 | 0.503 | 0.808 |
| L pars orbitalis | 0.136 | 0.713 | 0.919 | 0.744 | 0.389 | 0.977 | 0.193 | 0.661 | 0.838 |
| **L pars triangularis** | 0.087 | 0.769 | 0.928 | 0.025 | 0.874 | 0.977 | 5.915 | 0.016 | 0.303 |
| L middle frontal gyrus | 0.173 | 0.678 | 0.905 | 0.104 | 0.748 | 0.977 | 0.052 | 0.819 | 0.951 |

| | | | | | | | | | | |
|---|---|---|---|---|---|---|---|---|---|---|
| L superior frontal gyrus | 0.001 | 0.973 | 0.986 | | 0.025 | 0.874 | 0.977 | 8.542 | 0.004 | 0.126 |
| L long insular gyrus | 0.486 | 0.486 | 0.905 | | 0.012 | 0.914 | 0.979 | 1.209 | 0.273 | 0.808 |
| L short insular gyrus | 0.811 | 0.369 | 0.850 | | 2.741 | 0.099 | 0.977 | 0.784 | 0.377 | 0.808 |
| L middle occipital gyrus | 3.974 | 0.048 | 0.549 | MDD < HC | 1.611 | 0.206 | 0.977 | 2.051 | 0.154 | 0.678 |
| L superior occipital gyrus | 2.618 | 0.107 | 0.627 | | 0.222 | 0.638 | 0.977 | 2.236 | 0.136 | 0.678 |
| L lateral occipito-temporal gyrus | 0.007 | 0.933 | 0.958 | | 0.233 | 0.630 | 0.977 | 0.571 | 0.451 | 0.808 |
| L lingual gyrus | 0.361 | 0.549 | 0.905 | | 0.092 | 0.762 | 0.977 | 0.010 | 0.922 | 0.982 |
| L parahippocampal gyrus | 2.213 | 0.138 | 0.702 | | 0.699 | 0.404 | 0.977 | 0.633 | 0.427 | 0.808 |
| L orbital gyrus | 7.066 | 0.009 | 0.351 | MDD > HC | 0.072 | 0.789 | 0.977 | 0.010 | 0.921 | 0.982 |
| L angular gyrus | 6.914 | 0.009 | 0.351 | MDD > HC | 1.754 | 0.187 | 0.977 | 1.009 | 0.316 | 0.808 |
| **L supramarginal gyrus** | 0.010 | 0.920 | 0.958 | | 0.089 | 0.765 | 0.977 | 4.568 | 0.034 | 0.354 |
| **L superior parietal lobule** | 1.309 | 0.254 | 0.758 | | 0.099 | 0.754 | 0.977 | 1.938 | 0.166 | 0.678 |
| L postcentral gyrus | 0.380 | 0.538 | 0.905 | | 2.045 | 0.154 | 0.977 | 1.823 | 0.178 | 0.678 |
| L precentral gyrus | 0.000 | 0.991 | 0.991 | | 2.273 | 0.133 | 0.977 | 4.277 | 0.040 | 0.354 |
| L precuneus | 0.650 | 0.421 | 0.905 | | 0.002 | 0.961 | 0.987 | 0.582 | 0.446 | 0.808 |
| L straight gyrus | 0.012 | 0.913 | 0.958 | | 0.271 | 0.603 | 0.977 | 0.328 | 0.567 | 0.816 |
| L subcallosal gyrus | 4.818 | 0.029 | 0.549 | MDD > HC | 0.623 | 0.431 | 0.977 | 0.859 | 0.355 | 0.808 |
| L anterior transverse temporal gyrus | 0.090 | 0.764 | 0.928 | | 0.881 | 0.349 | 0.977 | 0.469 | 0.494 | 0.808 |
| L lateral superior temporal gyrus | 0.555 | 0.457 | 0.905 | | 0.000 | 0.992 | 0.992 | 0.290 | 0.591 | 0.816 |
| L planum polare | 0.025 | 0.874 | 0.958 | | 0.467 | 0.495 | 0.977 | 0.738 | 0.391 | 0.808 |
| L planum temporale | 1.341 | 0.248 | 0.758 | | 0.000 | 0.991 | 0.992 | 0.005 | 0.942 | 0.982 |
| L inferior temporal gyrus | 1.124 | 0.290 | 0.758 | | 1.124 | 0.290 | 0.977 | 0.102 | 0.750 | 0.920 |
| L middle temporal gyrus | 0.102 | 0.750 | 0.928 | | 1.035 | 0.310 | 0.977 | 0.001 | 0.982 | 0.982 |
| **R frontomarginal gyrus** | 0.058 | 0.810 | 0.947 | | 0.111 | 0.740 | 0.977 | 8.076 | 0.005 | 0.126 |

| | | | | | | | | | | |
|---|---|---|---|---|---|---|---|---|---|---|
| R inferior occipital gyrus | 0.042 | 0.838 | 0.958 | | 0.018 | 0.895 | 0.979 | | 0.833 | 0.363 | 0.808 |
| R paracentral lobule | 2.948 | 0.088 | 0.555 | | 0.007 | 0.932 | 0.979 | | 0.001 | 0.976 | 0.982 |
| **R subcentral gyrus** | 2.047 | 0.154 | 0.728 | | 8.608 | 0.004 | 0.167 | CC > CT + TT | 0.002 | 0.962 | 0.982 |
| R transverse frontopolar gyrus | 0.339 | 0.561 | 0.905 | | 0.117 | 0.732 | 0.977 | | 2.148 | 0.144 | 0.678 |
| R anterior cingulate gyrus | 0.274 | 0.601 | 0.905 | | 0.006 | 0.941 | 0.979 | | 0.077 | 0.782 | 0.944 |
| R anterior midcingulate gyrus | 0.264 | 0.608 | 0.905 | | 0.637 | 0.426 | 0.977 | | 1.116 | 0.292 | 0.808 |
| **R posterior midcingulate gyrus** | 0.189 | 0.664 | 0.905 | | 2.322 | 0.129 | 0.977 | | 4.973 | 0.027 | 0.354 |
| R dorsal posterior cingulate gyrus | 1.180 | 0.279 | 0.758 | | 0.102 | 0.750 | 0.977 | | 0.227 | 0.634 | 0.838 |
| R ventral posterior cingulate gyrus | 1.039 | 0.309 | 0.758 | | 0.423 | 0.516 | 0.977 | | 0.302 | 0.583 | 0.816 |
| R cuneus | 0.231 | 0.632 | 0.905 | | 0.322 | 0.571 | 0.977 | | 4.270 | 0.040 | 0.354 |
| R pars opercularis | 1.671 | 0.198 | 0.758 | | 0.460 | 0.498 | 0.977 | | 0.614 | 0.434 | 0.808 |
| R pars orbitalis | 0.239 | 0.626 | 0.905 | | 0.007 | 0.933 | 0.979 | | 0.872 | 0.352 | 0.808 |
| R pars triangularis | 0.566 | 0.453 | 0.905 | | 8.312 | 0.004 | 0.167 | CC < CT + TT | 0.041 | 0.840 | 0.951 |
| R middle frontal gyrus | 3.139 | 0.078 | 0.549 | | 0.223 | 0.638 | 0.977 | | 0.035 | 0.851 | 0.951 |
| R superior frontal gyrus | 0.325 | 0.569 | 0.905 | | 0.034 | 0.854 | 0.977 | | 8.610 | 0.004 | 0.126 |
| R long insular gyrus | 0.173 | 0.678 | 0.905 | | 0.774 | 0.380 | 0.977 | | 0.004 | 0.952 | 0.982 |
| R short insular gyrus | 0.016 | 0.901 | 0.958 | | 1.294 | 0.257 | 0.977 | | 0.199 | 0.656 | 0.838 |
| R middle occipital gyrus | 0.366 | 0.546 | 0.905 | | 0.239 | 0.625 | 0.977 | | 3.085 | 0.081 | 0.557 |
| R superior occipital gyrus | 0.097 | 0.756 | 0.928 | | 1.134 | 0.288 | 0.977 | | 0.013 | 0.909 | 0.982 |
| R lateral occipito-temporal gyrus | 0.032 | 0.858 | 0.958 | | 0.734 | 0.393 | 0.977 | | 2.564 | 0.111 | 0.649 |
| R lingual gyrus | 0.350 | 0.555 | 0.905 | | 1.411 | 0.236 | 0.977 | | 0.214 | 0.644 | 0.838 |
| R parahippocampal gyrus | 5.233 | 0.023 | 0.549 | MDD > HC | 0.759 | 0.385 | 0.977 | | 1.006 | 0.317 | 0.808 |
| R orbital gyrus | 0.014 | 0.905 | 0.958 | | 0.390 | 0.533 | 0.977 | | 1.362 | 0.245 | 0.808 |
| R angular gyrus | 3.257 | 0.073 | 0.549 | | 0.272 | 0.603 | 0.977 | | 0.376 | 0.541 | 0.808 |

| | | | | | | | | | | | |
|---|---|---|---|---|---|---|---|---|---|---|---|
| R supramarginal gyrus | 1.312 | 0.253 | 0.758 | | 0.592 | 0.443 | 0.977 | | 0.107 | 0.744 | 0.920 |
| R superior parietal lobule | 1.045 | 0.308 | 0.758 | | 2.655 | 0.105 | 0.977 | | 0.549 | 0.459 | 0.808 |
| R postcentral gyrus | 3.966 | 0.048 | 0.549 | MDD < HC | 0.868 | 0.353 | 0.977 | | 0.872 | 0.352 | 0.808 |
| R precentral gyrus | 0.159 | 0.691 | 0.905 | | 2.276 | 0.133 | 0.977 | | 2.662 | 0.104 | 0.649 |
| R precuneus | 0.310 | 0.579 | 0.905 | | 0.029 | 0.865 | 0.977 | | 0.489 | 0.485 | 0.808 |
| R straight gyrus | 1.515 | 0.220 | 0.758 | | 1.392 | 0.239 | 0.977 | | 2.390 | 0.124 | 0.672 |
| R subcallosal gyrus | 3.385 | 0.067 | 0.549 | | 0.355 | 0.552 | 0.977 | | 0.053 | 0.818 | 0.951 |
| R anterior transverse temporal gyrus | 1.509 | 0.221 | 0.758 | | 0.947 | 0.332 | 0.977 | | 0.413 | 0.521 | 0.808 |
| R lateral superior temporal gyrus | 0.301 | 0.584 | 0.905 | | 2.922 | 0.089 | 0.977 | | 0.426 | 0.515 | 0.808 |
| R planum polare | 0.299 | 0.585 | 0.905 | | 0.056 | 0.813 | 0.977 | | 0.318 | 0.573 | 0.816 |
| R planum temporale | 1.963 | 0.163 | 0.728 | | 1.010 | 0.316 | 0.977 | | 1.844 | 0.176 | 0.678 |
| R inferior temporal gyrus | 2.282 | 0.133 | 0.702 | | 0.332 | 0.565 | 0.977 | | 1.324 | 0.251 | 0.808 |
| R middle temporal gyrus | 0.161 | 0.689 | 0.905 | | 0.072 | 0.789 | 0.977 | | 1.007 | 0.317 | 0.808 |

The F and P values were obtained using analysis of covariance (ANCOVA) adjusted for age, gender, education level, medication, and total cortical surface area as covariates

The false discovery rate (FDR) was applied in each analysis for multiple comparison correction, q < 0.05; cortical surface area: 76 comparisons in both hemispheres.

*Regions that remained significant after the multiple comparison correction are marked with an asterisk.

Cortical regions which had significant diagnosis-by-genotype interaction or genotype effect in the main analysis on the genotype are represented with bolded-face.

MDD, major depressive disorder; HC, healthy controls; CC, CC genotype of rs1360780; CT + TT, CT or TT genotype of rs1360780; L, left hemisphere; R, right hemisphere. $P_{uncorr}$, uncorrected p-value; $P_{corr}$, FDR-corrected p-value.

Table S14. Exploratory analysis investigating correlations between cortical surface area and DNA methylation in intron 7 of the *FKBP5* gene among groups determined by diagnosis, genotype, and diagnosis-by-genotype interaction.

| | **Subgroups** | **Regions** | **r or t** | **$P_{uncorr}$** | **CpG** |
|---|---|---|---|---|---|
| Diagnostic groups | MDD (n=104) | L anterior midcingulate gyrus | 0.215 | 0.034 | pos 2 |
| | | L suparmarginal gyrus | -0.241 | 0.017 | pos 1 |
| | | R inferior occipital gyrus | 0.226 | 0.025 | pos 1 |
| | | R middle frontal gyrus | 0.281 | 0.005 | pos 1 |
| | | R subcallosal gyrus | -0.239 | 0.018 | pos 1 |
| | HC (n=82) | L frontomarginal gyrus | 0.242 | 0.034 | pos 1 |
| | | L orbital gyrus | 0.251 | 0.028 | pos 1 |
| | | R dorsal posterior cingulate gyrus | 0.233 | 0.041 | pos 2 |
| | | R pars orbitalis | 0.245 | 0.032 | pos 2 |
| | | R short insular gyrus | -0.300 | 0.008 | pos 1 |
| | | R parahippocampal gyrus | -0.267 | 0.019 | pos 2 |
| | | R straight gyrus | 0.229 | 0.046 | pos 1 |
| | | R lateral superior temporal gyrus | 0.229 | 0.045 | pos 2 |
| | | R planum temporale | 0.227 | 0.048 | pos 2 |
| Genotype groups | T (n= 68) | L anterior midcingulate gyrus | 0.333 | 0.008 | pos 2 |
| | | L posterior midcingulate gyrus | 0.271 | 0.033 | pos 2 |
| | | L ventral posterior cingulate gyrus | 0.272 | 0.033 | pos 1 |
| | | L fusiform gyrus | 0.344 | 0.006 | pos 2 |
| | | L supramarginal gyrus | -0.271 | 0.033 | pos 1 |
| | | L supramarginal gyrus | -0.389 | 0.002 | pos 2 |
| | | L precuneus | -0.260 | 0.042 | pos 2 |
| | | L planum polare | 0.253 | 0.048 | pos 2 |
| | | R dorsal posterior cingulate gyrus | -0.253 | 0.047 | pos 1 |
| | | R subcallosal gyrus | -0.314 | 0.013 | pos 2 |
| | CC (n= 118) | R subcentral gyrus | 0.214 | 0.024 | pos 1 |
| | | R short insular gyrus | -0.255 | 0.007 | pos 1 |
| | | R middle occipital gyrus | -0.228 | 0.015 | pos 1 |
| | | R superior occipital gyrus | -0.213 | 0.024 | pos 1 |
| | | R rectus gyrus | 0.193 | 0.041 | pos 1 |
| | | R planum temporale | 0.210 | 0.026 | pos 1 |

| | | | | | |
|---|---|---|---|---|---|
| Interaction effects | Diagnosis × methylation | R transverse frontopolar gyrus | 2.211 | 0.028 | pos 1 |
| | | R pars orbitalis | -2.215 | 0.028 | pos 2 |
| | | R middle frontal gyrus | 2.453 | 0.015 | pos 1 |
| | | R short insular gyrus | 2.562 | 0.011 | pos 1 |
| | | R parahippocampal gyrus | 2.079 | 0.039 | pos 2 |
| | Genotype × methylation | L middle frontal gyrus | -2.322 | 0.021 | pos 2 |
| | | L supramarginal gyrus | -3.277 | 0.001 | pos 2 |
| | | L superior parietal lobule | -2.080 | 0.039 | pos 2 |
| | | L precuneus | -2.894 | 0.004 | pos 2 |
| | | R frontomarginal gyrus | -2.289 | 0.023 | pos 1 |
| | | R subcentral gyrus | -2.753 | 0.007 | pos 1 |
| | | R dorsal posterior cingulate gyrus | -2.582 | 0.011 | pos 2 |
| | | R middle occipital gyrus | 2.054 | 0.041 | pos 1 |
| | | R angular gyrus | -2.055 | 0.041 | pos 2 |
| | | R precuneus | -2.088 | 0.038 | pos 2 |
| | | R straight gyrus | -2.396 | 0.018 | pos 1 |
| | | R subcallosal gyrus | -2.448 | 0.015 | pos 2 |
| | Diagnosis × genotype × methylation | L planum polare | -2.024 | 0.044 | pos 1 |
| | | L planum polare | -2.360 | 0.019 | pos 2 |
| | | R inferior occipital gyrus | -2.077 | 0.039 | pos 1 |
| | | R superior frontal gyrus | -2.041 | 0.043 | pos 1 |
| | | R precuneus | -2.010 | 0.046 | pos 2 |

A two-tailed Pearson's partial correlation was performed to analyze the correlations between the cortical surface area and percentage of *FKBP5* gene DNA methylation adjusting for following covariates according to each analysis: MDD group: age, gender, education level, medication, total intracranial cavity volume, and *FKBP5* genotype; HC group: age, gender, education level, total intracranial cavity volume, and *FKBP5* genotype; T or CC group: age, gender, education level, medication, total intracranial cavity volume, and diagnosis.

A hierarchical moderated regression analysis was performed to investigated the interactive effect of the DNA methylation with the diagnosis, genotype, or both diagnosis and genotype in terms of the correlation with the cortical surface area.

Only regions with statistically significant correlations are shown ($P_{uncorr} < 0.05$). No cortical regions remained significant after the multiple comparison correction ($P_{corr} > 0.05$).

The FDR was applied in each analysis for multiple comparison correction, q < 0.05; for diagnosis effect (MDD or HC group): 304 comparisons (=38 cortical regions x 2 hemispheres x 2 diagnosis groups x 2 CpG sites); genotype effect (T allele carrier or C homozygote group): 304 comparisons (=38 cortical regions x 2 hemispheres x 2 genotype groups x 2 CpG sites); and interactive effects (e.g. diagnosis-by-methylation, genotype-by-methylation, diagnosis-by-genotype-by-methylation): 456 comparisons (=38 cortical regions x 2 hemispheres x 3 interactions x 2 CpG sites).

The r-values for Pearson's partial correlation analyses and t-value for the hierarchical moderated regression analyses.

MDD, patients with major depressive disorder; HC, healthy controls; MDD-T, MDD patients with T allele of rs1360780; MDD-CC, MDD patients with CC genotype of rs1360780; L, left hemisphere; R, right hemisphere; $P_{uncorr,}$ uncorrected P value.

Table S15. Differences in DNA methylation in intron 7 of the *FKBP5* gene between groups determined by antidepressant treatment.

| CpG | Drug-naïve (%, n = 49) | ADT (%, n = 55) | **Drug-naïve vs. ADT** | |
|---|---|---|---|---|
| | | | F | p |
| pos 1 | 97.97 ± 2.02 | 97.36 ± 2.97 | 1.000 | 0.320 |
| pos 2 | 91.11 ± 2.66 | 90.18 ± 6.95 | 0.506 | 0.479 |

Data are mean ± standard deviation (%).

The F and P values were obtained using analysis of covariance (ANCOVA) adjusted for age and gender.

Drug-naïve, Drug-naïve MDD patients; ADT, MDD patients with antidepressant treatment.
